# Fibronectin containing alternatively spliced extra domain A interacts at the central and c-terminal domain of Toll-like receptor-4

**DOI:** 10.1038/s41598-022-13622-2

**Published:** 2022-06-11

**Authors:** Shubhangi Gupta, Azeem Ali, Saurabh Pandey, Imran A. Khan, Prem Prakash

**Affiliations:** 1 Department of Molecular Medicine, Jamia Hamdard, New Delhi, India; 2 Department of Biochemistry, Jamia Hamdard, New Delhi, India; 3 Department of Chemistry, Jamia Hamdard, New Delhi, India

**Keywords:** Cellular signalling networks, Cardiology, Diseases, Health care, Medical research, Molecular medicine

## Abstract

Extra domain A of cellular fibronectin (FN-EDA) is known to cause insulin resistance, atherosclerosis, tissue fibrosis, ischemic stroke and exaggerated myocardial reperfusion injury through Toll-like receptor 4 (TLR4). However, the FN-EDA-TLR4 interacting site is not well established. Therefore, in-silico approaches have been used to study FN-EDA and TLR4 interactions at the interface. In the present study, molecular docking studies of FN-EDA with TLR4-myeloid differentiation factor 2 (MD2) heterodimer have been performed to unravel the FN-EDA-TLR4 interacting sequence. Furthermore, the modulatory role of FN-EDA adjacent domains FNIII(11) and FNIII(12) on its interaction with TLR4-MD2 was investigated. The results show that FN-EDA interacting sequence “SPEDGIRELF” selectively interacts with TLR4 directly near its central and C-terminal domain region. The regulatory domains, FN type III 11 facilitate and 12 impede the FN-EDA-TLR4 interaction. Furthermore, the molecular dynamic simulation studies confirmed that FN-EDA forms a stable complex with TLR4-MD2 heterodimer. In conclusion, FN-EDA interacts and forms a stable complex through its “SPEDGIRELF” sequence at the central and C-terminal domain region of TLR4. The revelation of FN-EDA and TLR4 interacting sites may help design novel therapeutics for drug discovery research.

## Introduction

The cellular fibronectin type III domain consists of alternatively spliced extra domain A (FN-EDA), which is a 94-residue domain present between the 11th and 12th domain of the FN type III region^1^. Healthy individuals express minute quantities of FN-EDA, whereas in a diseased individual with conditions like atherosclerosis, ischemic strokes, diabetes, arterial thrombosis, etc., elevated levels of FN-EDA can be detected^2–6^.

Toll-like receptor-4 (TLR4) is a member of TLR type I transmembrane glycoprotein present on the cell surface of some leukocytes, which are further involved in innate immune responses^7^. FN-EDA is a well-known damage-associated molecular pattern that directly interacts with and activates TLR4, resulting in the initiation of the inflammatory signalling cascade. Activation of TLR4 further requires the engagement of its accessory protein myeloid differentiation factor-2 or lymphocyte antigen 96 (MD2), bound to TLR4 near its N-terminal region. It also harbours a ligand-binding pocket for lipopolysaccharide (LPS), Eritoran etc.^7,8^.

Activation of TLR4 by FN-EDA has been shown to cause disease progression and tissue damage^4,6,9,10^. In response to tissue injury, upregulation of FN-EDA results in adverse cardiac tissue remodelling after myocardial infarction^11^. FN-EDA has also been shown to cause insulin resistance by the reduction in glucose disposal rate through activation of TLR4^5^.

FN-EDA has been shown to promote chronic cutaneous fibrosis through TLR4 signalling^12^. With the activation of TLR4 expressed on the platelets, FN-EDA assists in thrombus formation, and also promotes platelet aggregation^6^. Furthermore, FN-EDA instigates a post-ischemic thrombo-inflammatory response aggravating myocardial reperfusion injury through TLR4 activation^10^.

FN-EDA is found to be elevated in the atherosclerotic patients arteries^13^. It has also been suggested that the progression of atherosclerosis is modulated by TLR4^14^. FN-EDA and TLR4 double knockout mice were found to be protected against plaque development and atherosclerosis progression^4^.

All the above studies have indicated that FN-EDA directly interacts with and activates TLR4 causing disease progression and tissue damage, still the interaction site of FN-EDA and TLR4 is not known. Therefore, interactions between FN-EDA and TLR4 need to be well established, to help discover novel therapeutics to block their interaction and thus prevent tissue damage and disease progression^15^.

In this article, we have inspected the possible interacting site of FN-EDA with the TLR4-MD2 heterodimer complex using in-silico approaches and mutations were carried out to validate the interaction. Furthermore, protein–protein interactions were carried out to unravel the FN-EDA interacting sequence (SLiM sequence) with TLR4-MD2 heterodimer. FN-EDA but not the other alternatively spliced variant from the same RNA transcript extra domain B (FN-EDB) is known to interact with TLR4^6,8^. Thus, to further validate our observation we performed the same in-silico approach to unravel the possible interaction between FN-EDB and TLR4.

## Results

### Molecular docking of FN-EDA and FN-EDB with TLR4-MD2 heterodimer and interfacial residue determination

To determine the possible interacting regions of mouse FN-EDA (mFN-EDA) with the mouse TLR4-MD2 (mTLR4-MD2) heterodimer complex, protein–protein docking was performed. The resolved crystal structure of the mTLR4-MD2 complex having PDB ID: 3VQ1 was selected to perform protein–protein docking with the predicted structure of mFN-EDA.

Since mFN-EDA has ~ 96.7% sequence identity with human FN-EDA (hFN-EDA)^16^, therefore, the structure of mFN-EDA was deduced from the resolved crystal structure of hFN-EDA (PDB ID: 1J8K). A total of 3 mutations were carried out in hFN-EDA (His44 to Arg44; Glu53 to Asp53; and, Thr87 to Ile87).

Protein–protein docking of mTLR4-MD2 was performed with mFN-EDA using RosettaDock^17–19^. The top 10 docking decoys (models) obtained were ranked based on their total score (Supplementary Table S1, S2, S3). From these 10 top scoring entries, the best 3 models of mTLR4-MD2-FN-EDA were selected according to the four interfacial criteria:—total score, I_sc (interface score), Fnat and fa_dun shown in Table 1. In addition to the mouse, docking was also performed for humans using hFN-EDA and hTLR4-MD2 (PDB ID: 3FXI), along with the docking of FN-EDB (PDB ID: 2MNU) with mTLR4-MD2 in RosettaDock (Table 1). The mouse and human TLR4-MD2-FN-EDA top-scoring model complexes had a docking score of − 700 kcal/mol and − 751.6 kcal/mol respectively, whereas the docking score of the mouse TLR4-MD2-FN-EDB model complex was about − 695 kcal/mol.

**Table 1:** FN-EDA and FN-EDB top 3 docked model complexes with TLR4-MD2 obtained from RosettaDock.

Complex	Model No	Total score	I_sc	Fnat	fa_dun
Mouse TLR4-MD2-FN-EDA complex	1	− 700	− 8.678	0.048	60.468
	2	− 698.7	− 8.210	0.540	61.055
	3	− 698.3	− 6.260	0.159	60.803
Human TLR4-MD2-FN-EDA complex	1	− 751.6	− 6.377	0.492	61.181
	2	− 750	− 5.440	0.119	61.143
	3	− 749.8	− 6.139	0.017	61.419
TLR4-MD2-FN-EDB complex	1	− 695.998	− 6.149	0.516	61.038
	2	− 695.946	− 7.064	0.500	60.77
	3	− 695.402	− 6.794	0.435	60.804

Total score, Overall energy of the docking complex in kcal/mol; I_sc, Interface energy, which is the sum of the energies of individual protein partners in isolation, subtracted from the total score of the docked complex; Fnat, It is the fraction of the native contacts recovered in the docked model; fa_dun, Probability of obtaining native-like side chain rotamers using Dunbrack’s statistics.

Proximal residues present within the 5 Å range at the TLR4-MD2 interface with FN-EDA were identified using the top-scoring mouse model, along with their atom distances using the UCSF Chimera^20^. This evaluation gave an idea of the probable interacting residues of FN-EDA with TLR4-MD2 complex in the interfacial area (Supplementary Table S4). Proximal (interfacial) residues of mFN-EDA were found to be near the concave surface of the central domain and C-terminal region of TLR4. Similarly, hTLR4-MD2-FN-EDA was found near the concave region of the TLR4 C-terminal (Supplementary Table S5). However, mTLR4-MD2-FN-EDB complexes were found near the convex region of the TLR4 C-terminal (Supplementary Table S6).

### Docking validation and root mean square deviation (RMSD) matrix

For docking validation, the top identified docking model of FN-EDA was used as an input (Supplementary Figure S7) for re-docking TLR4-FN-EDA with MD2 using RosettaDock. The top 10 decoys obtained (Supplementary Table S7) were compared with the previously obtained decoys based on the same criteria (total score, I_sc, Fnat and fa_dun).

Most of the top model conformations obtained from validation docking were found nearly similar to their reference model (used as input for re-docking) with root mean square (RMS) values less than 4 Å.

To filter out the suitable re-docked models, protein structure alignment was performed to determine the RMSD value for each model, keeping one model as a reference for the others. Out of the top 10 validation models, the models having the lowest RMSD values (< 1.5) were considered for further studies.

The decoy models 1, 2, 3 and 4 from mTLR4-MD2-FN-EDA (Supplementary Figure S10), models 1,2,4,7.8 and 9 from hTLR4-MD2-FN-EDA (Supplementary Table S8; Figure S11) and models 1, 2, 3, 4 and 5 from mTLR4-MD2-FN-EDB (Supplementary Table S9; Figure S12) were considered.

### Obtaining the lowest energy models

The lowest energy models of FN-EDA with TLR4-MD2 were determined from the above filtered low RMSD models. This was done to determine the Prime energies of the selected models using Schrodinger (Prime)^21,22^. Furthermore, the PIPER scores for the lowest Prime energy models of FN-EDA were generated in Schrödinger^23,24^, by considering TLR4-MD2 heterodimer and FN-EDA as receptor and ligand respectively (Table 2).

**Table 2:** Prime energies and PIPER scores of the low RMSD models obtained for mouse TLR4-MD2-FN-EDA docked complexes.

Model Name	Model no	Prime energy (kcal/mol)	PIPER pose score (kcal/mol)
Mouse TLR4-MD2-FN-EDA	Model 1	− 35,171.98	− 732.792
	Model 2	− 35,182.51	− 729.138
	Model 4	− 35,154.34	− 714.977

Prime Energy, Overall energy of the entire docked complex of TLR4-MD2-FN-EDA using Schrödinger Prime module; PIPER score, Pose score of the top scoring entry obtained for TLR4-MD2-FN-EDA docked complex from Schrödinger BioLuminate (performs protein–protein docking using PIPER program).

The lowest prime energy out of the 3 low energy mouse models was − 35,182.5 kcal/mol approximately with a PIPER score of − 729 kcal/mol. Similarly, for human models, the lowest prime energy was approximately − 36,126.8 kcal/mol with a PIPER score of about − 601 kcal/mol. On the other hand, the FN-EDB model had the lowest prime energy and PIPER score of about − 30,866 kcal/mol and − 221.5 kcal/mol respectively (Supplementary Table S10).

Since the prime energies and PIPER scores for both mouse and human FN-EDA models were found to be much lower as compared to FN-EDB models, this shows that TLR4-MD2 dimer forms more stable complexes with FN-EDA as compared to FN-EDB.

For a complex to be stable, the total energy of the bound complex needs to be less than the sum of the individual energies of the binding proteins. Therefore, the individual prime energies of TLR4, MD2 and FN-EDA were calculated.

The sum of the individual prime energies of the docking partners in the mTLR4-MD2-FN-EDA model complex was − 34,909.82 kcal/mol, which is higher than the prime energy of the combined complex − 35,182.5 kcal/mol (Table 3). Similarly in the human model, it was − 35,873.16 kcal/mol vs. − 36,126.8 kcal/mol (Supplementary Table S11). Conversely, in mice FN-EDB complex it was lower, − 35,796.8 kcal/mol vs. − 30,866 kcal/mol(Supplementary Table S12).

**Table 3:** Prime energies of the individual binding proteins along with their total prime energy in mouse.

Proteins	Prime energy (kcal/mol)
Mouse TLR4	− 25,878.42
Mouse MD2	− 5420.83
Mouse EDA	− 3610.57
Total Prime Energy	− 34,909.82

The above data indicate that FN-EDA both in mice and humans forms a stable complex with TLR4-MD2 but not with mFN-EDB.

### Determination of short linear interacting motif (SLiM) sequence at the interfacial area

**Figure 1:**
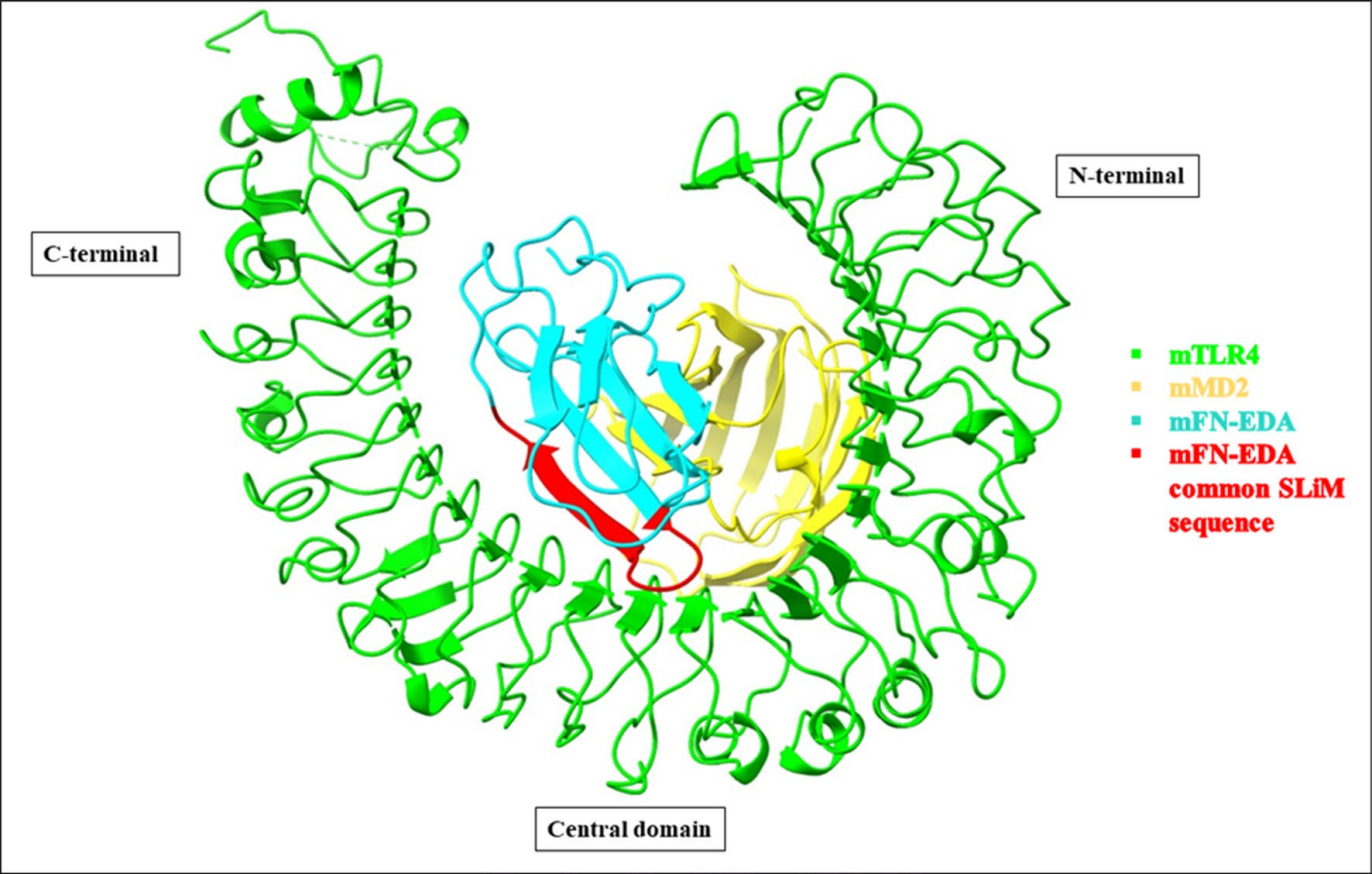
Representation of mouse TLR4-MD2-FN-EDA depicting the interacting peptide (SLiM sequence) of FN-EDA (“SPEDGIRELF”, highlighted in red) with the TLR4 in ChimeraX. All models of mouse FN-EDA SLiM sequences show interaction near the central and C-terminal region of TLR4.

Rosetta server^25,26^ was used to identify the SLiM sequence of FN-EDA interacting with TLR4. The receptor and partner chains were specified as TLR4-MD2, and FN-EDA respectively. In all the 3 low energy mTLR4-MD2-FN-EDA models, a common 10 amino acid FN-EDA peptide sequence “SPEDGIRELF” was found to have the highest contribution to the TLR4-FN-EDA interaction energy (highlighted red in Fig. 1) present in the C–C' domain whereas no FN-EDA interacting peptides were found with MD2. The total interface score, interface score and relative interface score of the above-determined mouse FN-EDA SLiM sequence was approximately − 19 REU, − 15 kcal/mol and 78% respectively (Table 4). Similar results were obtained in the human models "SPEDGIHELF" with -22 REU, − 9.7 kcal/mol and 51% respectively (Supplementary Table S13).

**Table 4:** SLiM sequences of mFN-EDA identified by Rosetta server (Peptiderive) for filtered TLR4-MD2-FN-EDA docked model complexes.

Model name	Model No	Receptor chain (mTLR4)	Partner chain (mFN-EDA)	Peptide sequence Position (Residue no.)	Best identified linear peptide sequence (SLiM sequence)	Interface score (kcal /mol)	Total interface score (REU)	Relative interface score (%)
Mouse TLR4-MD2-FN-EDA	1	A	B	38	SPEDGIRELF	− 14.954	− 19.26	77.64
	2	A	B	38	SPEDGIRELF	− 14.992	− 19.23	77.98
	3	A	B	38	SPEDGIRELF	− 15.045	− 19.34	77.81

Interface score, binding energy between the determined SLiM sequence of FN-EDA and TLR4 surface at the specified position; Relative interface score, percent contribution of the derived peptide in the interaction energy between TLR4 and FN-EDA; Total interface score, interaction energy of the entire TLR4 receptor-FN-EDA partner protein–protein complex in REU (Rosetta Energy units); Prime Energy, Overall energy of the protein complex in kcal/mol using Schrödinger Prime module.

Glu40 and Asp41 residues present in the C–C'-E FN-EDA domain are known to be majorly involved in maintaining the ideal conformation for antibody binding. Double mutation of Glu40 and Asp41 to Alanine had been shown to reduce antibody reactivity against FN-EDA^16^. Therefore, to observe the effects of this mutation on mTLR4-MD2-FN-EDA complex energy, a double mutation of Glu40 and Asp41 to Alanine was performed.

The prime energy of mTLR4-MD2 in complex with the double mutant mFN-EDA was increased to − 35,100.91 kcal/mol from − 35,182.5 kcal/mol (Table 5). Similarly in the human model, the prime energy increased to − 36,002.8 kcal/mol from − 36,126.8 kcal/mol (Supplementary Table S16). Furthermore, each residue in the SLiM sequence mutated to Alanine at a time through residue scanning showed drastic changes in both stability and prime energy with the maximum in Arg44 mutation (Supplementary Table S15; Figure S26). Accordingly, in the human model, major changes in prime energy and stability were observed in Asp41 and Phe47 respectively (Supplementary Table S17; Figure S27). The prime energy of the mTLR4-MD2-FN-EDA complex was increased from − 35,182.5 kcal/mol to − 34,207.66 kcal/mol when the FN-EDA SLiM sequence was fully mutated to Alanine (Table 5). A similar mutation in the human FN-EDA SLiM sequence also increased the complex prime energy from − 36,126.8 kcal/mol to − 35,706.6 kcal/mol (Supplementary Table S16).

**Table 5:** Prime energies of the mouse FN-EDA model complexes obtained after mutations in SLiM sequence.

Species model	Mutations	Prime energy (kcal/mol)
Mouse TLR4-MD2-FN-EDA	Glu40 + Asp41 to Alanine	− 35,100.91
	Whole SLiM sequence mutation (“SPEDGIRELF”)	− 34,207.66

### Significance of FN-EDA adjacent segments in its interaction with TLR4

In the fibronectin structure, FN-EDA is present between the FNIII(11) and FNIII(12) domains^1,27^. These domains have been suspected to modulate the FN-EDA activity towards the TLR4-MD2 complex. FNIII(11) domain enhances its activity whereas the FNIII(12) domain suppresses its activity towards TLR4-MD2 activation^1^.

To monitor the effects of the adjacent domains the TLR4-MD2 complex was docked with two separate joint segments FNIII(11)-EDA and FNIII(11)-EDA-(12) in HDOCK^28–30^. The crystal structure of the human FNIII(11)-EDA-(12) segment was available (PDB ID: 6XAX), but the mouse FNIII(11)-EDA-(12) segment was not. Therefore, a total of 21 mutations (7 in the hFNIII(11) domain, 3 in hFN-EDA, and 11 in the FNIII(12) domain) were carried out. These mutations were based on the local sequence alignment performed between human FNIII(11)-EDA-(12) and mouse fibronectin peptide sequence (NCBI; accession no.: NP_034363.1).

The resultant FNIII(11)-EDA mouse segment docking with TLR4-MD2 (Supplementary Table S18) gave a similar SLiM sequence as obtained solely with mFN-EDA "SPEDGIRELF" with a low interface score of − 13.797 kcal/mol with TLR4. However, mouse FNIII(11)-EDA-(12) segment docking with TLR4-MD2 gave a different SLiM sequence "TPTSFTAQWI" identified in mouse FNIII(12) domain with a higher interface score of − 6.417 kcal/mol with TLR4. On the other hand, in the human model, both segments gave a similar SLiM sequence as identified solely with hFN-EDA "SPEDGIHELF" but the interface score of hFNIII(11)-EDA-(12) was higher (− 4.360 kcal/mol) than the interface score of FNIII(11)-EDA (− 17.229 kcal/mol) with TLR4 (Supplementary Table S19).

The observation thus suggests that in both mice and humans, FNIII(11) domain supports whereas FNIII(12) domain may hinder FN-EDA interaction with TLR4.

### Identification of protein–protein interactions at the interface with FN-EDA

**Figure 2:**
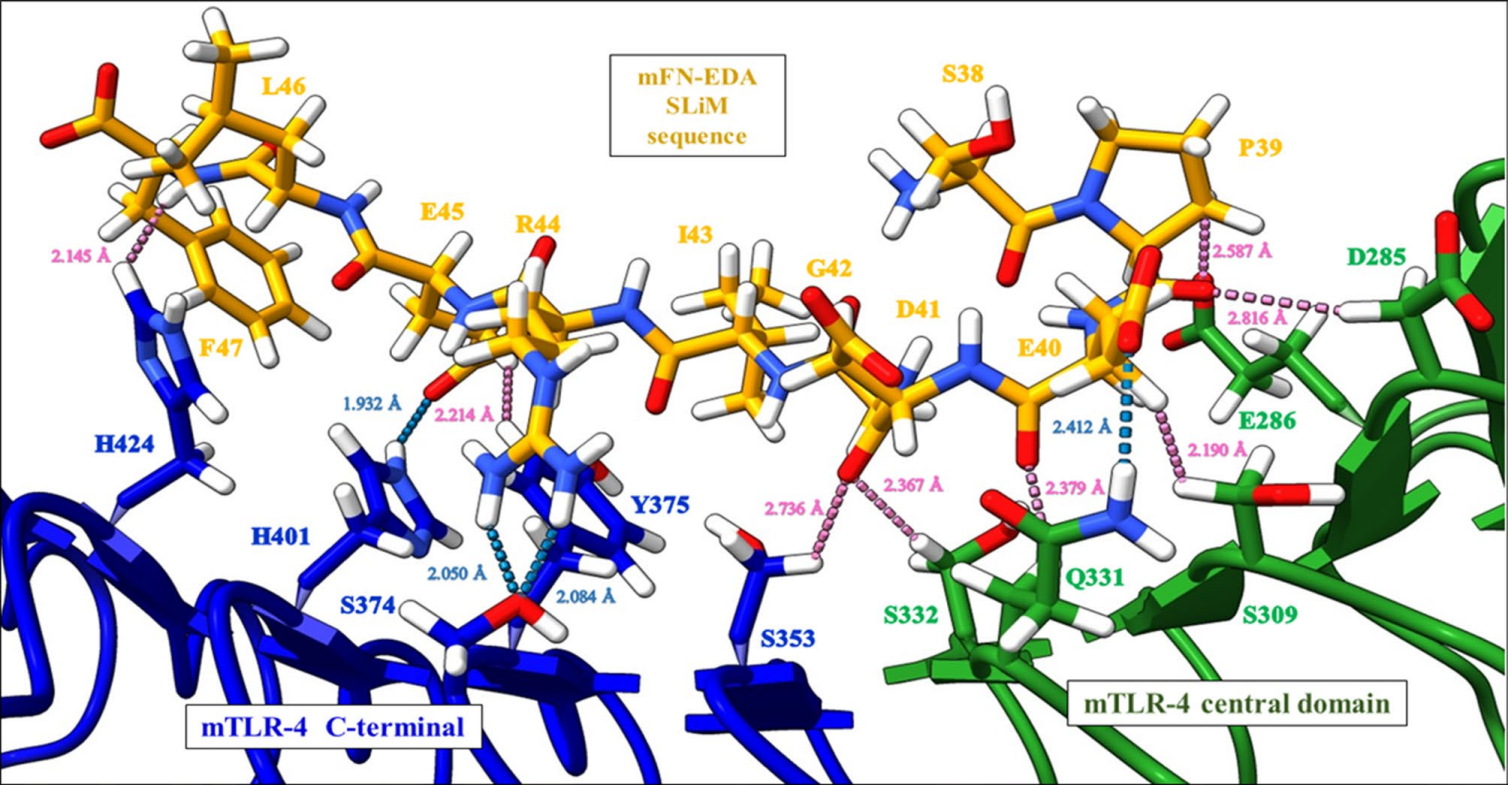
Protein–protein interactions of mouse FN-EDA SLiM sequence (orange sticks) with mTLR4 central domain (green sticks) and C-terminal (blue sticks) identified using ChimeraX^47,48^, showing hydrogen bonding (blue dashes) and Van-der-Waal interactions (pink dashes). mFN-EDA interacts with the residues of the central and C-terminal domains of mTLR4.

Protein–protein interactions were studied between the identified FN-EDA SLiM sequence and TLR4. The residues showing H-bonds and Van-der-Waal interactions between mFN-EDA and mTLR4 are shown in Fig. 2. TLR4 interacting residues include Asp285, Glu286, Ser309, Gln331, Ser332, Ser353, Ser374, Tyr375, His 401 and His 424 and FN-EDA interacting residues in the SLiM sequence include Pro39, Glu40, Asp41, Arg44, Glu45 and Phe47. A total of four H-bonds were formed, two between Arg44 and Ser374 (2.050 Å, 2.084 Å), one between Glu45 and Tyr375 (2.214 Å) and one between Glu45 and His 401 (1.932 Å). The distance considered to study the following interactions was below 3 Å (Supplementary Table S20). These interactions were also observed in the 2D ligand interaction diagram of the FN-EDA SLiM sequence with TLR4 in Schrödinger (Supplementary Figure S16).

Since drastic stability and prime energy changes were observed in the Arg44 residue mutation to Alanine in mFN-EDA, the interactions of this mutated SLiM sequence (SPEDGI ‘A’ELF) with TLR4 were studied using a 2D ligand interaction diagram. As a result, fewer interactions were observed, since Ala44 showed no interactions as compared to the two H-bonds formed by Arg44 with Ser374 (Supplementary Figure S28).

### Selective interaction of FN-EDA with TLR4-MD2 heterodimer

**Figure 3:**
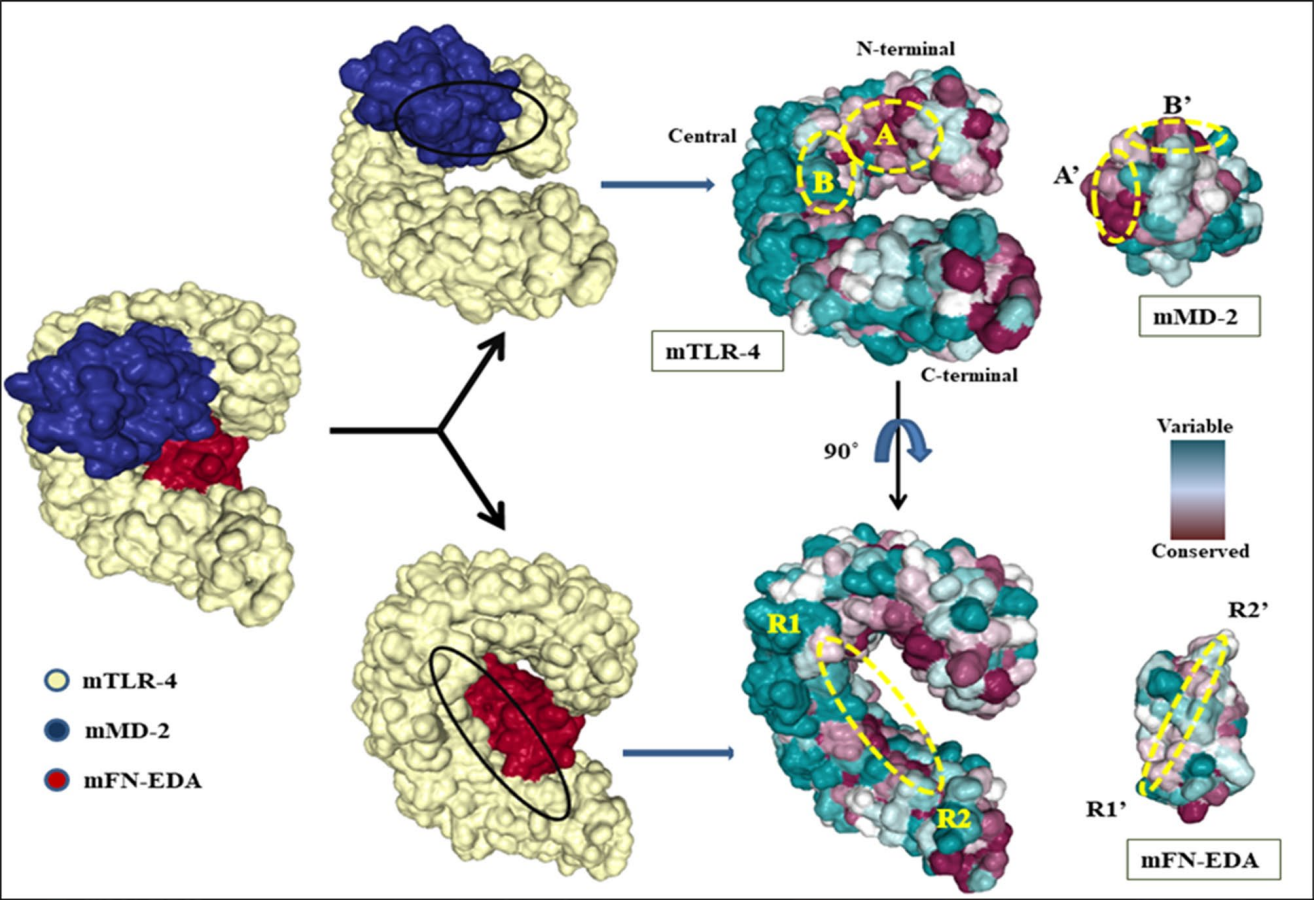
Representation of conserved regions in mouse TLR-4, MD-2 and FN-EDA according to the conservation scores obtained by the Bayesian method, using the ConSurf web server. Circular patches represent the interaction sites of MD-2 and FN-EDA with TLR-4 individually. Most of the residues in TLR-4, at the MD-2 interaction site, are strictly conserved, whereas most of the residues at the MD-2 interaction site are variable.

MD2 protein is known for its stable binding with TLR4 near the 'A' patch of the N-terminal domain with highly conserved residues and the 'B' patch of the central domain with residues showing a hypervariable region. Negatively charged A patch and positively charged B patch of TLR4 interact with positively charged A' and negatively charged B' sites of MD2 respectively, by charge complementarity resulting in stable interactions between TLR4 and MD2^7^. FN-EDA (R1'-R2' region) has shown interactions with TLR4 near the central (R1) and C-terminal domain (R2) region (Fig. 3). To determine if FN-EDA interacts with TLR4 at the selective region (variable region) or the non-selective region (conserved regions) of TLR4, the ConSurf web server^31,32^ was utilized, which estimated the conserved and non-conserved residues of TLR4 on the scale of 0–9. Residue conservation scores from 1 to 4 were considered as variable (cyan) whereas residues having conservation scores from 5 to 9 were considered as conserved (magenta).

In the mice model, 7 out of 10 TLR4 interacting residues with FN-EDA were found to be variable (Supplementary Table S21). Similarly in the human model, 6 out of 9 TLR4 interacting residues with FN-EDA were variable (Supplementary Table S22). Therefore, FN-EDA binds to the variable region of TLR4 in both mice and humans.

### Molecular dynamics (MD) simulation

Desmond Molecular dynamics simulations^33^ were carried out for the predicted mouse and human TLR4-MD2-FN-EDA models. This helped determine the stability of these complexes and the conformational changes taking place during the simulation time by keeping them in a simple point charge(SPC) water solvent system (Supplementary Figure S29).

**Figure 4:**
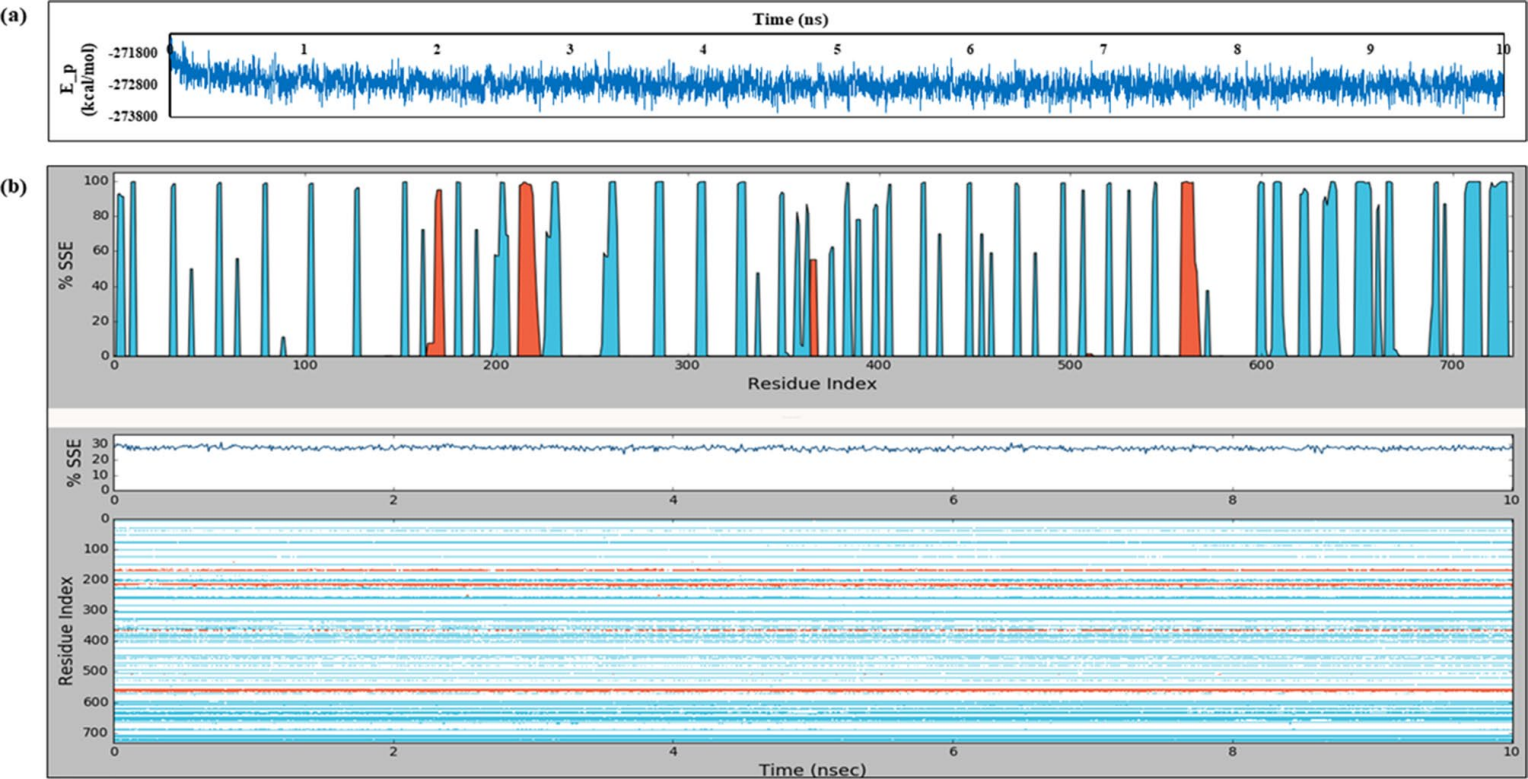
Desmond MD simulation outputs of mouse TLR4-MD2-FN-EDA (**a**) E_p (potential energy) plot vs simulation time; (**b**) TLR4-MD2 secondary structure changes during simulation where light blue refers to β-strands and orange colour refers to α-helices.

After the relaxation of FN-EDA model systems, MD simulations were carried out for 10 ns at constant pressure (1 atm) and temperature (300 K). The recording intervals for energy and trajectory were set as default 1.2 picoseconds and 10 picoseconds respectively. Throughout the simulation run, the potential energy for the mFN-EDA models was observed to be stable with an average value of − 272,785.457 kcal/mol (Supplementary Table S23). The potential energy (E_p) plot for mTLR4-MD2-FN-EDA against a simulation time of 10 ns is depicted in Fig. 4a. mTLR4-MD2 secondary structure elements (SSE) alpha-helices (orange) and beta-strands (blue) distribution during the simulation time are shown in Fig. 4b.

**Figure 5:**
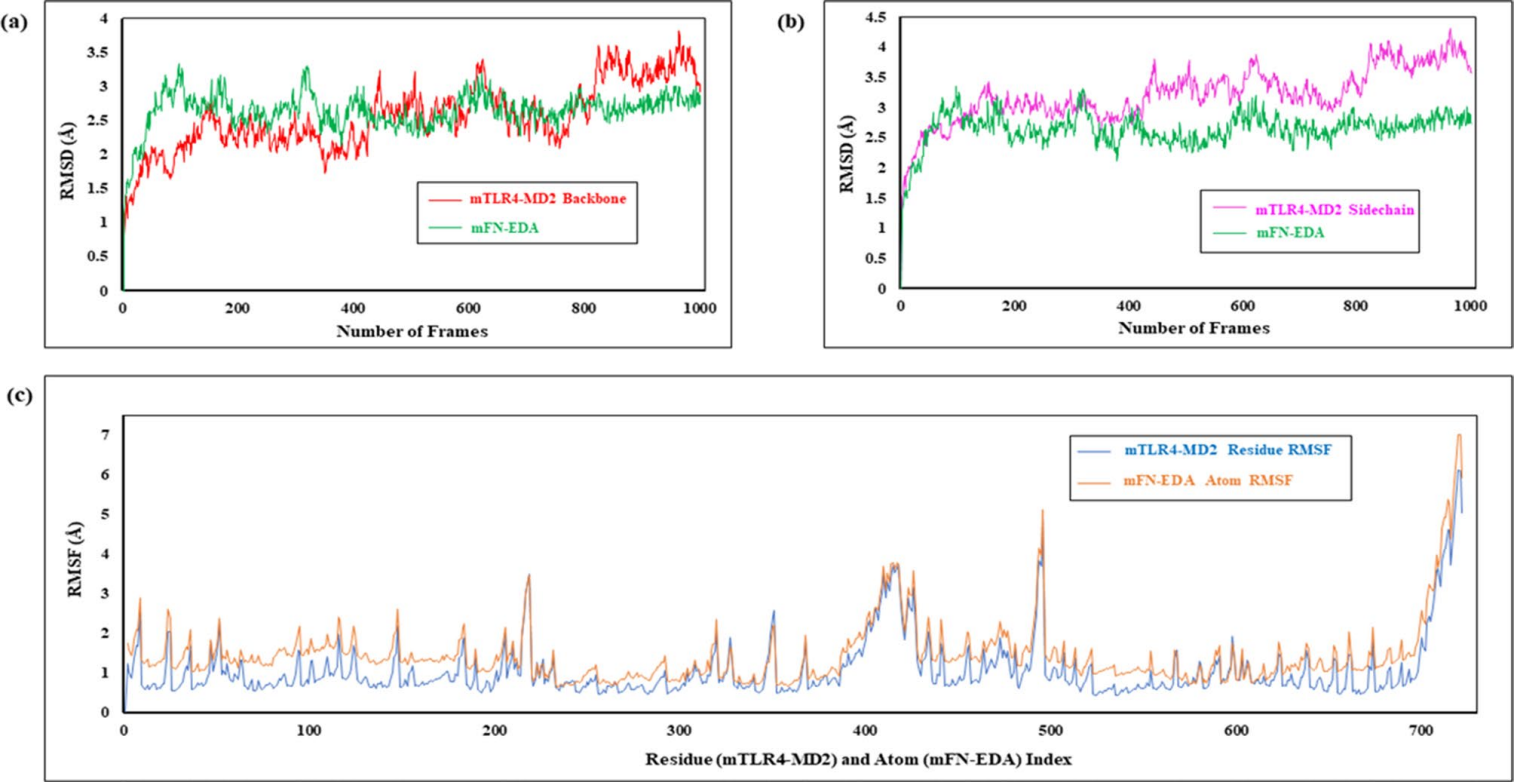
RMSD and RMSF plots of mouse TLR4-MD2-FN-EDA using Desmond Simulation Interactions Diagram. (**a**) TLR4-MD2 Backbone RMSD plot with FN-EDA; (**b**) TLR4-MD2 Sidechain RMSD plot with FN-EDA; (**c**) Backbone RMSF plot.

The RMSD plots generated for the mTLR4-MD2-FN-EDA model were depicted as the RMSD (Å) recorded after every 10 picoseconds of trajectory interval within 10 ns of simulation time (i.e., approximately 1000 frames generated). The backbone and sidechain RMSD plots of mTLR4-MD2-FN-EDA showed TLR4-MD2 and FN-EDA maintaining a plateau of equilibrium (Fig. 5a,b). After ~ 8 ns, the backbone and sidechain RMSD values of TLR4-MD2 are above 3 Å indicating the possibility of undergoing large conformational changes.

Backbone root mean square fluctuation(RMSF) plots generated for the FN-EDA showed major fluctuations with the C-terminal region of TLR4 and MD2. Individually, RMSF plots of mTLR4 (Residue number 1 to 596) and mMD2 (Residue number 597 to 731) showed fluctuations above 5 Å, whereas mFN-EDA showed fluctuations as high as 6 Å (Fig. 5c). Similarly, molecular dynamics of the human model (Supplementary Figure S30) show RMSD values of TLR4-MD2 above 4 Å, indicating its major conformational changes and the FN-EDA RMSD maintaining equilibrium with the TLR4-MD2 complex. On the other hand, molecular dynamics of the TLR4-MD2 and mFN-EDA (Arg44 mutation to Alanine) RMSD plot indicate the mutated complex to be a rigid structure as compared to the non-mutated mTLR4-MD2-FN-EDA model (Supplementary Figure S31). The radius of gyration (Rg) plots of the TLR4-MD2-FN-EDA complex had Rg of 30.04 Å vs. 29.58 Å higher than Arg44 mutated complex showing that the mutated complex is more compact than the non-mutated complex (Supplementary Figure S32).

## Discussion

In this report, we have proposed fibronectin FN-EDA, but not FN-EDB, interacts with the active site of Toll-like Receptor 4 (TLR4). We determined the docking and interfacial score for mouse TLR4-MD2-FN-EDA complexes through RosettaDock. Determination of RMSD enabled us to filter the most precise probable docking models, for which Prime energy and PIPER scores were evaluated using Schrödinger Maestro. Afterwards, the interacting peptide sequence (SLiM sequence) of FN-EDA with TLR4 was determined along with their specified interacting position using the Rosetta server (Peptiderive). We also validated the FN-EDA SLiM sequence by mutations and studied the role of FNIII(11) and FNIII(12) in FN-EDA interaction with TLR4-MD2 complex individually using HDOCK and Rosetta server. Desmond Molecular dynamics simulations were performed to study the interaction behaviour and stability of FN-EDA with the TLR4-MD2 complex. For overall structural validation of the findings in mouse models, the following procedures were also performed for human FN-EDA models and mouse FN-EDB.

To obtain optimal results, the 3-dimensional monomer form of the TLR4-MD2 complex used here was taken from the previous validated structural data of the LPS-bound mouse TLR4-MD2 dimerized structure^34^. RosettaDock optimized the rigid-body and side-chain conformations to return atomically accurate low energy docked structure complexes of monomer TLR4-MD2 complex and FN-EDA. FN-EDA has more stimulatory effects on TLR4 than that of FN-EDB^8^, which was justified by the docking results obtained by us in both mouse and human models, specifying more significantly favourable interactions of FN-EDA, rather than FN-EDB with TLR4-MD2 complex. Re-docking of MD2 with TLR4-FN-EDA complex was performed in RosettaDock for validation of the docking models obtained and to enhance the probability to identify near-native complexes.

RMSD of the generated docked models enabled the identification of the best docking models obtained which are identified based on their RMSD value. Models which have lower RMSD values (approaching '0') are said to be good docking solutions. Prime energy describes the total energy of the receptor protein complex so the lowest energy docked models were identified from the RMSD filtered models. The TLR4-MD2-FN-EDA combined complex energy in both mice and humans was lower than the sum of individual energies of the docking partners which signifies that FN-EDA spontaneously interacts with the TLR4-MD2 to form a stable complex. But on the other hand, TLR4-MD2-FN-EDB combined complex energy was higher than the sum of their energies indicating FN-EDB may not form a stable complex with TLR4-MD2. Our data is in corroboration the previous finding showing that FN-EDB may not interact with TLR4^8^. PIPER program returns a sufficient number of near-native conformations of the docked poses through Fast-Fourier Transformation (FFT) based algorithm thereby reducing the number of falsely deduced high scoring models.

Rosetta server (Peptiderive) used for identifying FN-EDA SLiM sequence with TLR4 returned the best linear interacting proteins. A common SLiM sequence was found in mouse “SPEDGIRELF” and human “SPEDGIHELF” from the selected model respectively. The obtained SLiM sequence resides in the C–C'-E domain previously shown to interact with the antibodies IST-9, DH1 and 3E2^16^. Moreover, the “EDGIHEL” motif sequence in FN-EDA C–C' loop has been shown to interact with integrins α9β1, α4β1 and α4β7^35–39^. The α4β1 integrin acts together with TLR4 to upregulate fibrotic NF-κB gene expression in response to FN-EDA^40^. This indicates that the same specified FN-EDA motif interacting with integrins may also be interacting with TLR4. Glu40 and Asp41 in the C–C'-E loop maintain the optimal structural configuration of FN-EDA for antibody binding^16^. Thus to validate our proposed model we performed a double mutation of Glu40 and Asp41 to Alanine in both mice and humans. Indeed the energy of the TLR4-MD2-FN-EDA complex increased considerably following the mutation confirming our finding in corroboration with the previously published report. We further tested our model by performing point mutation of each residue in the FN-EDA SLiM sequence to Alanine. As expected the TLR4-MD2-FN-EDA complex showed a marked change in the stability and prime energy highest with Arg44 in mFN-EDA.

FN-EDA is known to interact with TLR4 to promote a signalling cascade^4–6,9,12^. The mouse and human FN-EDA SLiM sequence identified in each model was found to be interacting near the TLR4 central and C-terminal domain, which implies the presence of direct interactions between FN-EDA and TLR4. LPS binds in the MD2 pocket, while FN-EDA directly interacts with TLR4, to trigger TLR4-MD2 based inflammatory signalling cascade^8^. The presence of LPS antagonist Eritoran (E5564) has blocking effects on LPS activation towards TLR4, whereas the activity of FN-EDA towards TLR4 remains unaffected. This suggests that FN-EDA mediated TLR4 activation is LPS independent^8^. Our ConSurf observation confirmed that FN-EDA selectively binds to TLR4. ConSurf identified the conserved and variable residues based on conservation scores for each residue. Most of the residues of TLR4 involved in protein–protein interactions with FN-EDA are part of the variable region in both mice and humans.

FNIII(11) domain enhances whereas FNIII(12) domain represses TLR4 activation by FN-EDA^1^. The HDOCK docking results show that in presence of FNIII(11) no change was observed in the FN-EDA SLiM sequence that contributes to the binding energy and directly interacts with TLR4 whereas when FNIII(12) is added to the FNIII(11)-EDA segment the SLiM sequence was different in the mouse. Regardless of no change in the SLiM sequence in humans, the interaction of the SLiM sequence of FN-EDA with TLR4 is highly reduced. A fast and robust protein–protein docking program, HDOCK performs fast and robust protein–protein docking, predicting suitable interactions between macromolecules^29^. The finding was further validated by Schrödinger BioLuminate.

Desmond Molecular dynamics (MD) simulations helped in studying the stability and residual behaviour of TLR4-MD2-FN-EDA complexes. We have further validated our observation by mutating mFN-EDA Arg44 to Alanine which made the TLR4-MD2-FN-EDA complex more compact as confirmed by Rg values^41^.

FN-EDA deficiency has shown better survival rates following myocardial infarction^15^. Survival within subjects can also be improved by building ways to block interactions between TLR4 and FN-EDA. This is the first report to identify specific interacting sites between FN-EDA and TLR4 that can be therapeutically targeted to block the crosstalk. To make them further considerable, wet lab studies are required for detailed information about FN-EDA and TLR4 interactions and to validate the results obtained in the given study.

## Conclusion

FN-EDA interact with TLR4 around the central and the C-terminal domain which is affected by a point or whole mutation of the interacting site. FN-EDA and TLR4-MD2 heterodimer conformations may be more favourable than FN-EDB and TLR4-MD2. FNIII(11) enhances but FNIII(12) domain restricts FN-EDA binding to TLR4.

## Methodology

### Molecular docking and validation

The crystal structures of mouse and human TLR4-MD2 dimer, FN-EDA and FN-EDB were retrieved from RCSB PDB and prepared using Schrödinger ^42^. Mouse FN-EDA was derived from human FN-EDA by mutation of 3 residues in Schrӧdinger. RosettaDock (Docking2) was used to obtain suitable docked models of TLR4-MD2-FN-EDA and re-docking them for validation. RosettaDock used supercomputers to perform local docking between the specified proteins in the submitted complex for docking.

A total of 1000 decoys (models) were generated, of which the top 10 were classified based on the total score and I_sc (interface score). Of these 10 decoys, 3 decoys were considered for FN-EDA complexes based on total score, F-nat, I_sc and fa_dun). The total score defines the overall energy score (docking score) of the protein complex, whereas I_sc defines the interfacial score between the docked proteins in the complex (total score minus the sum of individual protein partners in isolation). Fnat represents the defined residue-residue contacts across the interfacial area whereas fa_dun represents the probability of observing native-like side-chain rotamers given by Dunbrack's statistics^43^.

### Proximal interfacial residues and their atom distances

UCSF Chimera was used to identify the proximal residues (within 5 Å zone selection) of FN-EDA with TLR4-MD2 heterodimer. The atom distances between these proximal residues were also calculated for FN-EDA complexes at the TLR-4 interface separately.

### RMSD matrix, Prime energy and PIPER scores

RMSD values for docked models were determined through protein structure alignment using Schrödinger, taking each model as a reference. Using these values an RMSD matrix was formed to identify low RMSD models.

The energies of each model of FN-EDA were calculated using Schrödinger (Prime), defined as Prime energy, to select the lowest energy models. PIPER pose score was determined for the lowest Prime energy models of FN-EDA using protein–protein docking through Schrödinger BioLuminate. PIPER module^23,24^ use FFT based approach to return near-native conformations of the docking poses. The ligand (mFN-EDA) rotated 70,000 times at every 5˚ angle, which returned the top 30 lowest energy stable complexes of mTLR4-MD2-FN-EDA. The decoy models selected were checked using PROCHECK^44^ (Supplementary Figure S13), which enabled to check the quality of these decoy models.

### Determining interacting peptide sequence

FN-EDA structure is comprised of seven β-strands denoted by A, B, C, C', E, F and G in a sequence^16^. Rosetta server (Peptiderive) was used for FN-EDA models to obtain the best short linear peptide (SLiM sequence) of FN-EDA interacting with TLR4 at the TLR4-FN-EDA interface which is mostly responsible for its contribution to their interaction energy. The obtained SLiM sequence was present in the C–C' domain of FN-EDA. The major criteria taken into consideration for the identification of the best short linear peptide included the total interface score in REU (Rosetta Energy Units) which is the binding energy of the receptor-partner complex; interface score which corresponds to the receptor-peptide (of partner chain) binding energy; and the relative interface score which describes the per cent contribution of the peptide in the interaction energy with the receptor.

The changes in stability and prime energy of the overall FN-EDA complex with TLR4-MD2 due to mutation of each residue in the SLiM sequence were performed using Residue scanning (Schrödinger).

### FN-EDA SLiM sequence validation using FNIII(11) and FNIII(12) domains

The SLiM sequence of FN-EDA was verified by adding FNIII(11) and FNIII(12) domains to FN-EDA, creating FNIII(11)-FN-EDA and FNIII(11)-EDA-(12) segments and docking them with TLR4-MD2 separately in HDOCK. HDOCK returned the top 10 best-docked models according to their docking score, along with the residues present at the interface. The mutations to be made in human FNIII(11)-EDA-(12) (PDB ID: 6XAX) to obtain mouse FNIII(11)-EDA-(12) segment, were identified using EMBOSS Matcher^45,46^. Emboss Matcher is a pairwise local sequence alignment tool which provides information about the sequence similarity and identity between two given sequences. The protein-peptide interactions were studied between the FN-EDA SLiM sequence and TLR4 using UCSF Chimera X^47,48^.

### Determining FN-EDA selectivity towards TLR4

The protein sequences of FN-EDA, TLR4 and MD2 were used for the determination of conserved and variable residues using the ConSurf server to explain the selective binding of FN-EDA on a defined TLR4 region. To identify conservation scores for each residue in the input protein sequence, ConSurf used the UNIREF-90 protein database to perform an HMMER homology search algorithm with an E-value cutoff of 0.0001 using 150 sequences. The maximum per cent identity and minimum per cent homology was used as default, i.e., 95% and 35% respectively. The multiple sequence alignment was performed using ClustalW and the calculations were performed using the Bayesian method.

### Molecular dynamics

Desmond molecular dynamics simulations for the TLR4-MD2-FN-EDA model complexes were performed by building a system for each FN-EDA model using a 10 × 10 × 10 (Å) buffer distance orthorhombic box of the SPC solvent system and neutralizing the system by adding NaCl under the OPLS_2005 force field. After system building, model system regeneration was performed. Molecular dynamics simulations were performed for 10 ns under a constant temperature of 300 K and pressure of 1 atm, with ensemble class as NPT.

The plots and average potential energy value were analysed using the Desmond Simulation Quality Analysis tab, and the RMSD plots of protein (TLR4-MD2) and ligand (FN-EDA); Protein (TLR4-MD2) and Ligand (FN-EDA) RMSF plots; and Protein (TLR4-MD2) SSE of all the TLR4-MD2-FN-EDA model complexes were studied using the Simulation Interaction Diagram (SID) tool of Desmond. The Rg plots were obtained using Desmond Simulation Event analysis (SEA).

## Supplementary Information

Supplementary Information.

## Data Availability

All the relevant data is provided within the manuscript and the supplementary material. Validated protein structures from RCSB PDB (https://www.rcsb.org) were used as a primary data source. The PDB IDs used here for TLR4-MD2 complex, FN-EDA and FN-EDB were 3FXI, 3VQ1, 1J8K and 2MNU.
