# Supplementary material for: Fibronectin containing alternatively spliced extra domain A interacts at the central and c-terminal domain of Toll-like receptor-4
Source: Sci Rep. 2022 Jun 11;12:9662. doi: 10.1038/s41598-022-13622-2 (PMC9188610; doi:10.1038/s41598-022-13622-2)
Supplement: Supplementary file 1 — Supplementary Information. [file 41598_2022_13622_MOESM1_ESM.docx]

**SUPPLEMENTARY MATERIAL**

**Primary docking results from RosettaDock**

The top 10 obtained models of mouse TLR4-MD2-FN-EDA, human TLR4-MD2-FN-EDA and mouse TLR4-MD2-FN-EDB submitted complexes in RosettaDock are given below (Table S1, S2 and S3 respectively). The top 10 poses along with their total score vs RMSD plots are also shown in the figure below (Figure S1, S2 for mouse; S3, S4 for human; and S5, S6 for FN-EDB).

**Table S1: RosettaDock output table of mouse TLR4-MD2 dimer docked with FN-EDA**

| **decoy** | **proteins_0286** | **proteins_0117** | **proteins_0626** | **proteins_0176** | **proteins_0639** | **proteins_0717** | **proteins_0304** | **proteins_0315** | **proteins_0731** | **proteins_0665** |
| --- | --- | --- | --- | --- | --- | --- | --- | --- | --- | --- |
| **total_score** | -700 | -699.4 | -698.7 | -698.3 | -697.5 | -697.3 | -696.8 | -696.4 | -696.3 | -696.2 |
| **rms** | 17.052 | 52.041 | 4.484 | 18.355 | 59.153 | 14.517 | 51.78 | 8.028 | 53.934 | 30.301 |
| **Fnat** | 0.048 | 0 | 0.54 | 0.159 | 0 | 0.286 | 0 | 0.19 | 0 | 0.032 |
| **I_sc** | -8.678 | -8.338 | -8.21 | -6.26 | -5.875 | -5.68 | -2.979 | -3.412 | -4.277 | -4.479 |
| **Irms** | 6.752 | 14.763 | 1.63 | 5.154 | 19.003 | 2.987 | 16.964 | 3.913 | 15.763 | 9.064 |
| **cen_rms** | 15.428 | 51.099 | 5.526 | 7.005 | 57.457 | 11.762 | 52.18 | 9.135 | 51.643 | 12.767 |
| **dslf_ca_dih** | 1.649 | 1.649 | 1.649 | 1.649 | 1.649 | 1.649 | 1.649 | 1.649 | 1.649 | 1.649 |
| **dslf_cs_ang** | 2.662 | 2.662 | 2.662 | 2.662 | 2.662 | 2.662 | 2.662 | 2.662 | 2.662 | 2.662 |
| **dslf_ss_dih** | 41.781 | 41.781 | 41.781 | 41.781 | 41.781 | 41.781 | 41.781 | 41.781 | 41.781 | 41.781 |
| **dslf_ss_dst** | -12.13 | -12.13 | -12.13 | -12.13 | -12.13 | -12.13 | -12.13 | -12.13 | -12.13 | -12.13 |
| **fa_atr** | -1258 | -1259 | -1261 | -1257 | -1253 | -1254 | -1254 | -1253 | -1254 | -1252 |
| **fa_dun** | 60.468 | 60.295 | 61.055 | 60.803 | 60.761 | 60.576 | 60.523 | 60.332 | 60.674 | 60.44 |
| **fa_elec** | -8.908 | -9.073 | -8.981 | -8.911 | -8.979 | -8.86 | -8.911 | -8.974 | -8.9 | -8.931 |
| **fa_pair** | -21.24 | -20.72 | -20.65 | -21.33 | -20.11 | -19.85 | -20.77 | -20.15 | -21.39 | -19.88 |
| **fa_rep** | 56.616 | 57.972 | 58.652 | 56.883 | 56.421 | 57.348 | 56.833 | 57.001 | 57.159 | 57.403 |
| **fa_sol** | 521.93 | 522.2 | 522.31 | 521.65 | 519.3 | 518.1 | 519.74 | 518.8 | 520.54 | 517.22 |
| **hbond_bb_sc** | -14.54 | -14.46 | -14.65 | -14.49 | -14.96 | -14.69 | -14.66 | -14.38 | -14.68 | -14.52 |
| **hbond_lr_bb** | -45.76 | -45.76 | -45.76 | -45.76 | -45.76 | -45.76 | -45.76 | -45.76 | -45.76 | -45.76 |
| **hbond_sc** | -9.227 | -9.27 | -8.64 | -8.984 | -9.185 | -8.448 | -8.586 | -8.478 | -8.673 | -8.531 |
| **hbond_sr_bb** | -15.42 | -15.42 | -15.42 | -15.42 | -15.42 | -15.42 | -15.42 | -15.42 | -15.42 | -15.42 |
| **interchain_contact** | -19 | -20 | -20 | -20 | -3 | -20 | -20 | -11 | -6 | -4 |
| **interchain_env** | -82.37 | -86.07 | -88.98 | -88.2 | -84 | -84.29 | -86.31 | -85.04 | -88.31 | -86.17 |
| **interchain_pair** | -0.853 | 2.401 | 0.917 | -1.314 | 1.874 | -3.419 | 1.026 | 2.752 | 2.601 | 1.461 |
| **interchain_vdw** | 0.196 | 0.253 | 0.972 | 0 | 0.452 | 0.785 | 0.003 | 0 | 0.172 | 0.129 |
| **st_rmsd** | 15.676 | 51.483 | 8.335 | 6.335 | 58.756 | 8.913 | 52.195 | 9.436 | 52.195 | 12.765 |


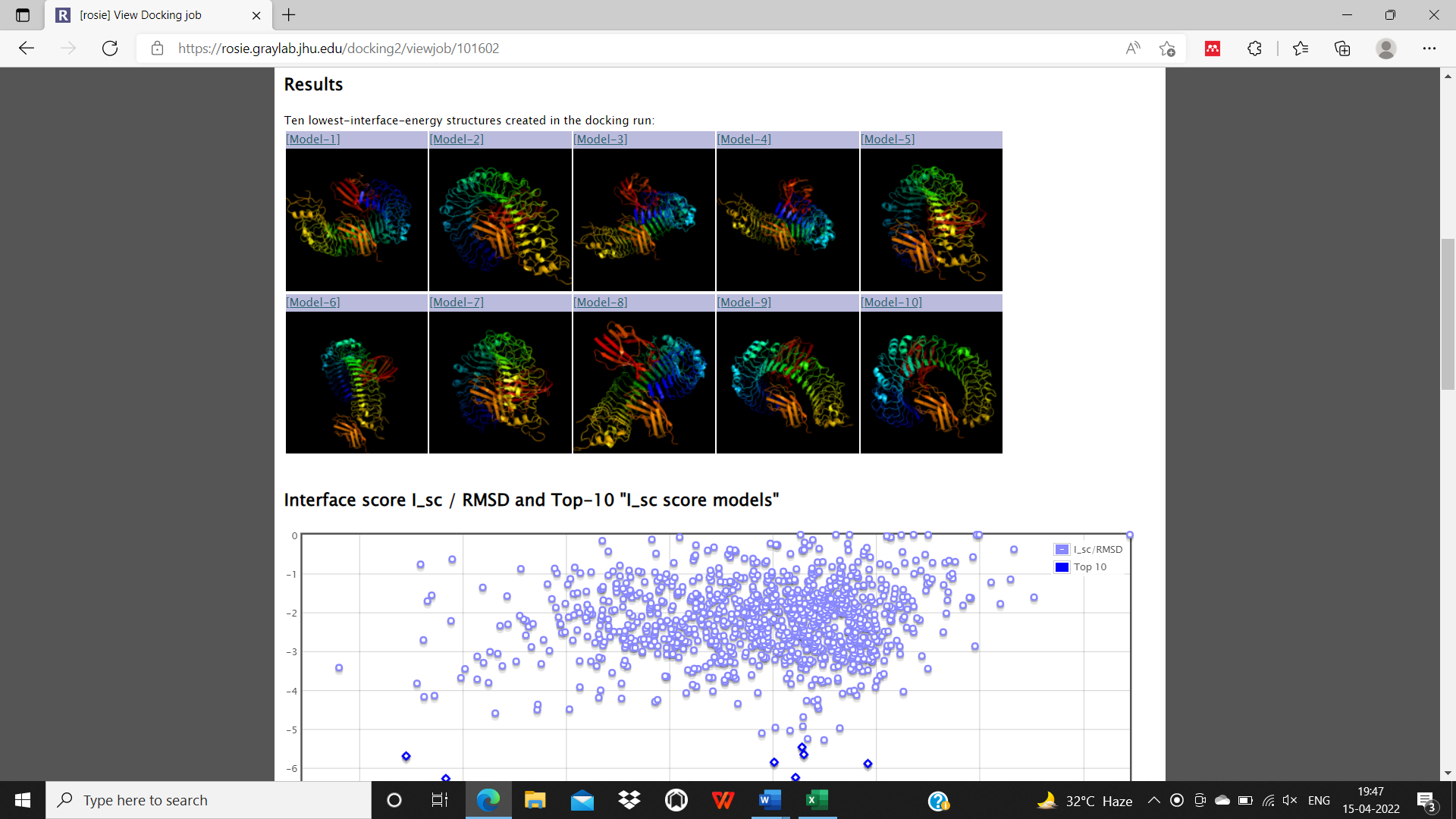


**Figure S1: Top 10 lowest interfacial energy of mTLR-MD2 docked structure with mFN-EDA by RosettaDock**


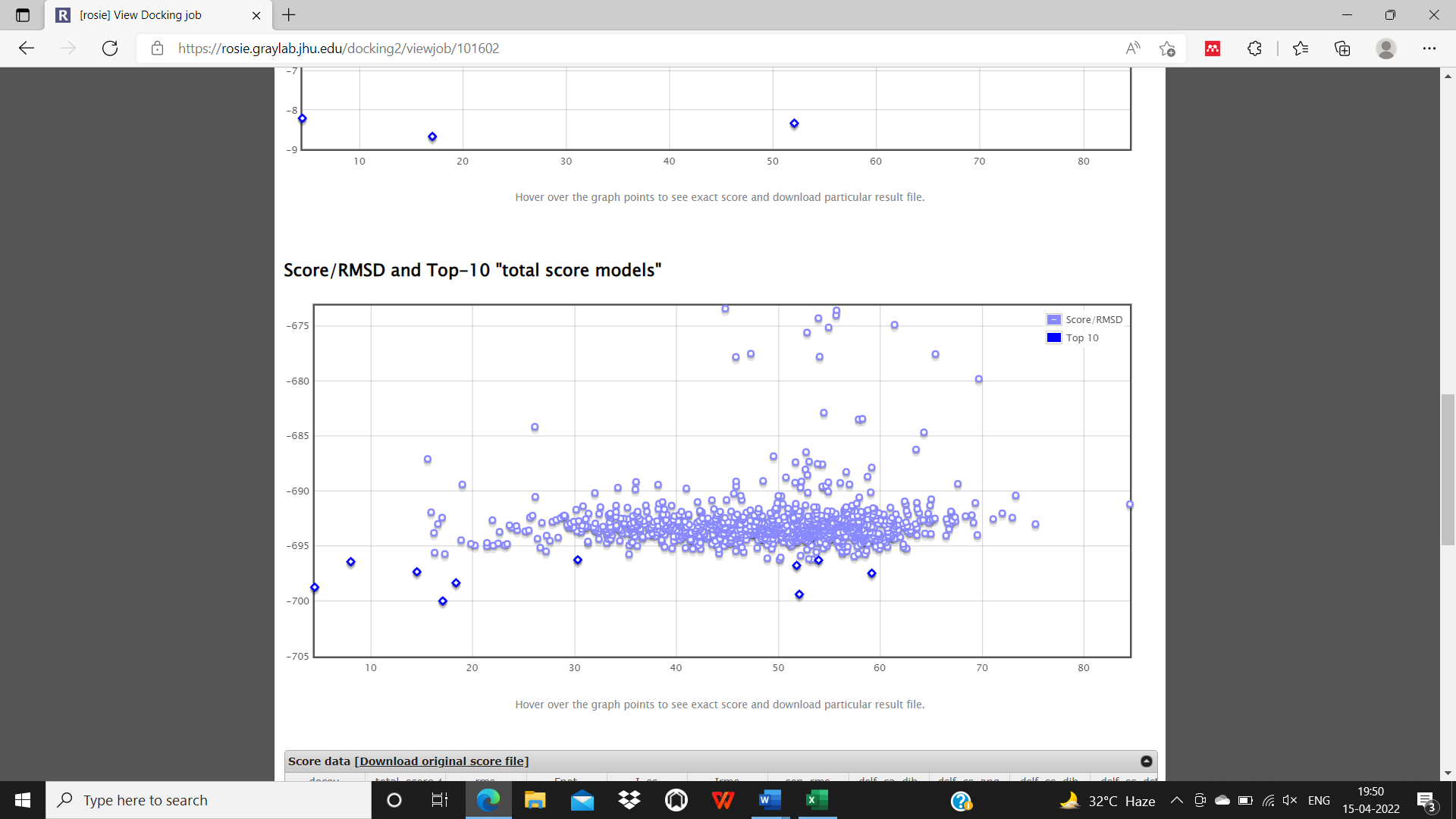


**Figure S2: The score v/s RMSD plot of 1000 docked mTLR4-MD2-FN-EDA models generated by RosettaDock, from which top 10 low scoring models are highlighted in dark blue**

**Table S2: RosettaDock output table of human TLR4-MD2 dimer docked with FN-EDA**

| **decoy** | **proteins_0342** | **proteins_0591** | **proteins_0017** | **proteins_0830** | **proteins_0973** | **proteins_0035** | **proteins_0118** | **proteins_0480** | **proteins_0513** | **proteins_0667** |
| --- | --- | --- | --- | --- | --- | --- | --- | --- | --- | --- |
| **total_score** | -751.6 | -750.9 | -750.8 | -750.2 | -750 | -749.8 | -749.4 | -749.4 | -749.4 | -749 |
| **rms** | 7.311 | 31.882 | 25.877 | 35.285 | 8.871 | 23.845 | 9.987 | 29.124 | 5.941 | 53.343 |
| **Fnat** | 0.492 | 0 | 0 | 0 | 0.119 | 0.017 | 0.424 | 0 | 0.508 | 0 |
| **I_sc** | -6.377 | -5.718 | -6.294 | -4.612 | -5.44 | -6.139 | -5.795 | -4.484 | -6.449 | -3.667 |
| **Irms** | 1.752 | 9.252 | 7.932 | 10.122 | 4.341 | 8.732 | 2.814 | 8.667 | 1.323 | 15.79 |
| **cen_rms** | 6.655 | 33.095 | 26.527 | 29.351 | 12.933 | 22.876 | 10.534 | 17.63 | 4.881 | 53.011 |
| **dslf_ca_dih** | 0.607 | 0.607 | 0.607 | 0.607 | 0.607 | 0.607 | 0.607 | 0.607 | 0.607 | 0.607 |
| **dslf_cs_ang** | 2.962 | 2.962 | 2.962 | 2.962 | 2.962 | 2.962 | 2.962 | 2.962 | 2.962 | 2.962 |
| **dslf_ss_dih** | 3.81 | 3.81 | 3.81 | 3.81 | 3.81 | 3.81 | 3.81 | 3.81 | 3.81 | 3.81 |
| **dslf_ss_dst** | -13.86 | -13.86 | -13.86 | -13.86 | -13.86 | -13.86 | -13.86 | -13.86 | -13.86 | -13.86 |
| **fa_atr** | -1249 | -1248 | -1254 | -1244 | -1248 | -1247 | -1249 | -1244 | -1246 | -1245 |
| **fa_dun** | 61.181 | 60.552 | 62.307 | 60.942 | 61.143 | 61.419 | 61.301 | 60.438 | 61.251 | 60.958 |
| **fa_elec** | -8.69 | -8.705 | -8.863 | -8.561 | -8.608 | -8.737 | -8.7 | -8.734 | -8.605 | -8.637 |
| **fa_pair** | -20.64 | -21.28 | -21.5 | -21.34 | -21.03 | -21.76 | -21.5 | -21.18 | -21.14 | -20.62 |
| **fa_rep** | 43.999 | 43.482 | 45.614 | 43.144 | 43.933 | 43.522 | 45.482 | 43.287 | 45.127 | 43.395 |
| **fa_sol** | 509.74 | 511.14 | 514.59 | 508.04 | 510.28 | 510.81 | 510.78 | 508.47 | 507.61 | 508.55 |
| **hbond_bb_sc** | -15.34 | -15.4 | -15.53 | -15.25 | -15.37 | -15.3 | -15.44 | -15.09 | -15.23 | -15.2 |
| **hbond_lr_bb** | -43.78 | -43.56 | -43.56 | -43.56 | -43.56 | -43.56 | -43.56 | -43.56 | -43.56 | -43.56 |
| **hbond_sc** | -10.48 | -10.49 | -10.88 | -10.83 | -10.54 | -10.94 | -10.52 | -10.75 | -10.84 | -10.57 |
| **hbond_sr_bb** | -12.03 | -12.03 | -12.03 | -12.03 | -12.03 | -12.03 | -12.03 | -12.03 | -12.03 | -12.03 |
| **interchain_contact** | -20 | -20 | -20 | -20 | -12 | -20 | -20 | -6 | -18 | -11 |
| **interchain_env** | -80.39 | -84.36 | -82.99 | -85.4 | -82.61 | -84.65 | -90.17 | -89.3 | -80.85 | -81.55 |
| **interchain_pair** | 0.021 | -0.804 | -1.99 | -0.202 | 0.861 | 0.413 | -0.581 | 0.176 | 1.462 | -2.171 |
| **interchain_vdw** | 0.015 | 0.023 | 0.351 | 0.022 | 0.997 | 0.528 | 0.362 | 0.595 | 0.032 | 0.213 |
| **st_rmsd** | 5.164 | 33.189 | 15.75 | 27.69 | 13.068 | 20.458 | 7.549 | 14.067 | 7.893 | 53.394 |


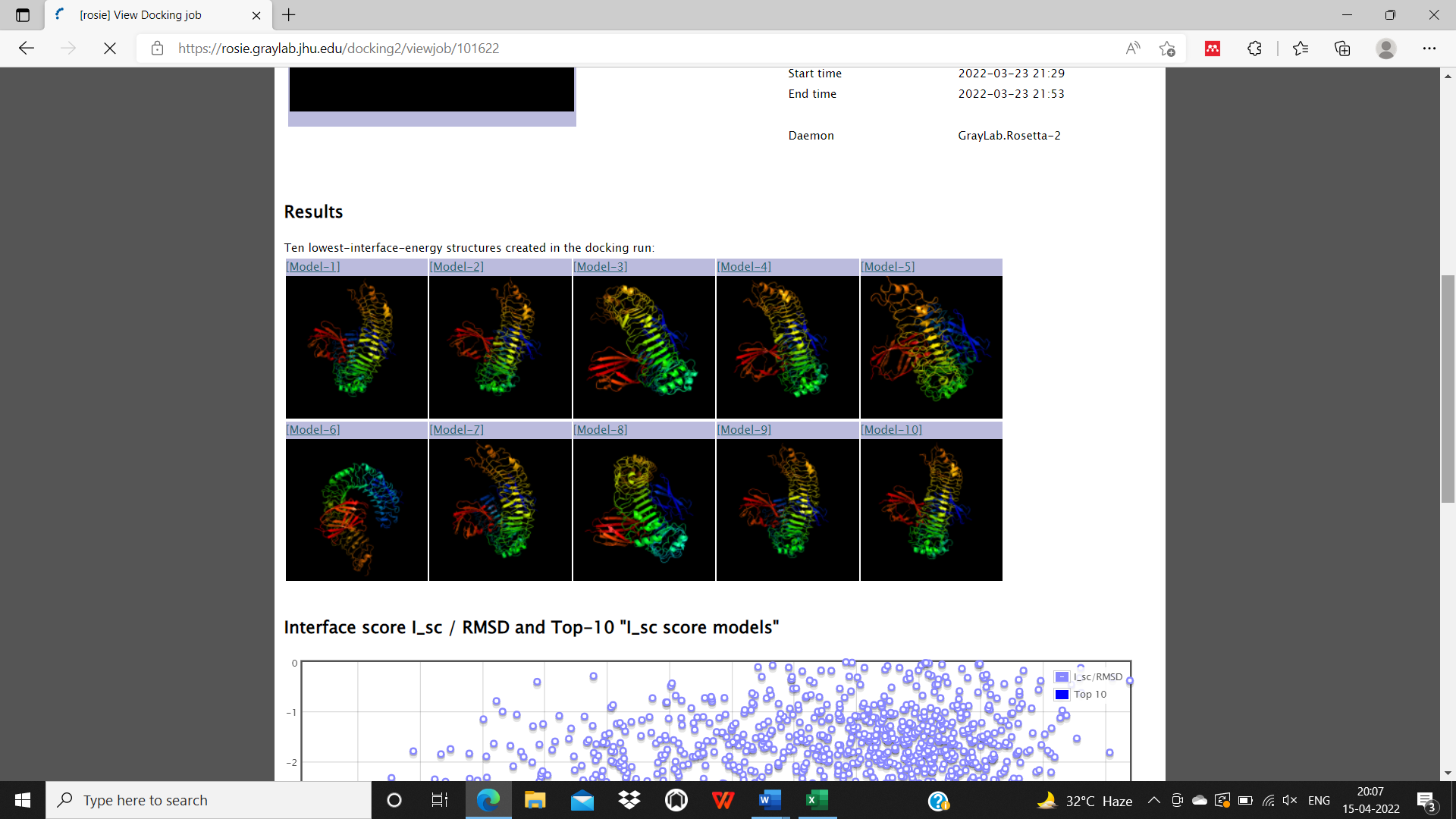


**Figure S3: Top 10 lowest interfacial energy of hTLR-MD2 docked structure with hFN-EDA by RosettaDock**


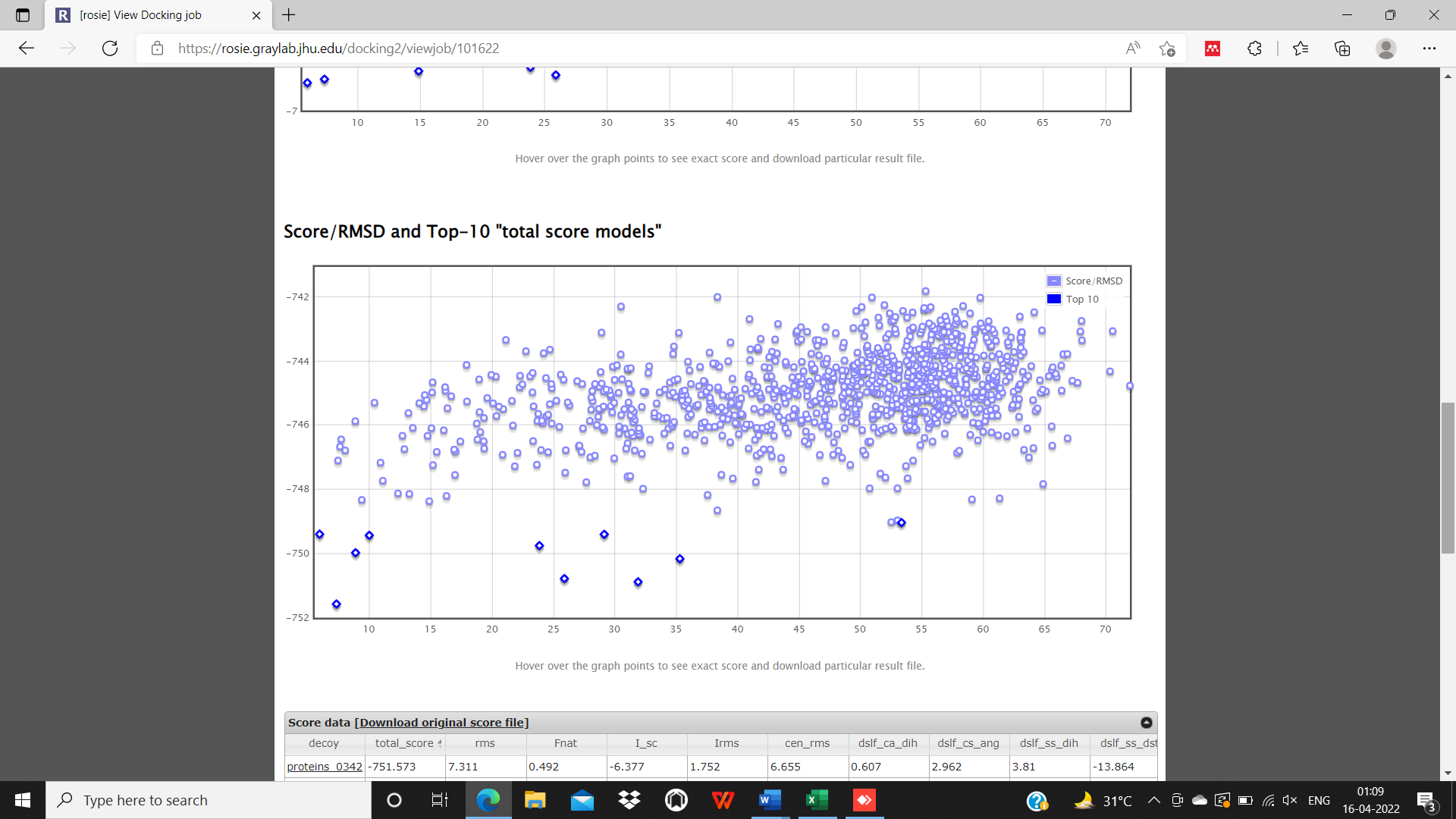


**Figure S4: The score v/s RMSD plot of 1000 docked hTLR4-MD2-FN-EDA models generated by RosettaDock, from which top 10 low scoring models are highlighted in dark blue**

**Table S3: RosettaDock output table of mouse TLR4-MD2 dimer docked with FN-EDB**

| **decoy** | **proteins_0986** | **proteins_0566** | **proteins_0164** | **proteins_0130** | **proteins_0065** | **proteins_0867** | **proteins_0257** | **proteins_0026** | **proteins_0098** | **proteins_0067** |
| --- | --- | --- | --- | --- | --- | --- | --- | --- | --- | --- |
| **total_score** | -695.998 | -695.946 | -695.402 | -695.382 | -695.018 | -694.851 | -694.345 | -694.01 | -693.881 | -693.64 |
| **rms** | 4.428 | 10.604 | 9.161 | 16.228 | 2.923 | 12.24 | 8.203 | 16.053 | 9.127 | 10.055 |
| **Fnat** | 0.516 | 0.5 | 0.435 | 0.161 | 0.516 | 0.306 | 0.194 | 0.032 | 0.21 | 0.177 |
| **I_sc** | -6.149 | -7.064 | -6.794 | -8.112 | -5.446 | -6.406 | -5.86 | -5.139 | -6.177 | -4.783 |
| **Irms** | 1.425 | 1.693 | 1.952 | 3.388 | 1.202 | 3.071 | 3.2 | 4.994 | 3.183 | 3.276 |
| **cen_rms** | 9.656 | 2.626 | 8.429 | 16.651 | 6.634 | 5.711 | 8.352 | 11.926 | 6.838 | 11.527 |
| **dslf_ca_dih** | 1.644 | 1.644 | 1.644 | 1.644 | 1.644 | 1.644 | 1.644 | 1.644 | 1.644 | 1.644 |
| **dslf_cs_ang** | 2.661 | 2.661 | 2.661 | 2.661 | 2.661 | 2.661 | 2.661 | 2.661 | 2.661 | 2.661 |
| **dslf_ss_dih** | 41.759 | 41.759 | 41.759 | 41.759 | 41.759 | 41.759 | 41.759 | 41.759 | 41.759 | 41.759 |
| **dslf_ss_dst** | -12.195 | -12.195 | -12.195 | -12.195 | -12.195 | -12.195 | -12.195 | -12.195 | -12.195 | -12.195 |
| **fa_atr** | -1261.808 | -1262.826 | -1261.891 | -1264.245 | -1261.912 | -1260.655 | -1262.684 | -1257.858 | -1259.492 | -1258.368 |
| **fa_dun** | 61.038 | 60.77 | 60.804 | 61.45 | 61.251 | 61.182 | 60.942 | 61.14 | 61.04 | 61.182 |
| **fa_elec** | -9.055 | -8.937 | -8.909 | -9.028 | -9.061 | -9.08 | -9.087 | -8.936 | -8.999 | -9.073 |
| **fa_pair** | -20.838 | -20.486 | -20.527 | -21.793 | -20.557 | -20.821 | -20.983 | -20.28 | -20.652 | -20.73 |
| **fa_rep** | 62.549 | 62.938 | 62.717 | 63.574 | 62.22 | 62.185 | 62.946 | 62.013 | 62.866 | 62.33 |
| **fa_sol** | 523.954 | 523.987 | 524.187 | 526.676 | 525.2 | 524.626 | 527.219 | 522.241 | 523.042 | 523.516 |
| **hbond_bb_sc** | -15.908 | -15.705 | -15.561 | -15.798 | -15.973 | -15.59 | -15.849 | -15.818 | -15.61 | -16.094 |
| **hbond_lr_bb** | -45.512 | -45.512 | -45.512 | -45.512 | -45.512 | -45.512 | -45.512 | -45.512 | -45.512 | -45.512 |
| **hbond_sc** | -8.78 | -8.537 | -9.072 | -9.068 | -9.038 | -9.547 | -9.7 | -9.363 | -8.927 | -9.253 |
| **hbond_sr_bb** | -15.508 | -15.508 | -15.508 | -15.508 | -15.508 | -15.508 | -15.508 | -15.508 | -15.508 | -15.508 |
| **interchain_contact** | -14 | -15 | -20 | -20 | -20 | -15 | -20 | -4 | -20 | -20 |
| **interchain_env** | -83.893 | -85.523 | -87.793 | -83.423 | -85.755 | -84.953 | -81.73 | -88.44 | -81.532 | -81.617 |
| **interchain_pair** | -0.996 | -1.46 | -0.8 | -1.149 | -0.732 | -0.931 | -1.045 | 0.092 | -4.62 | -4.14 |
| **interchain_vdv** | 0.005 | 0.389 | 0.216 | 0 | 0.01 | 0 | 0.363 | 0.61 | 0.219 | 0 |
| **st_rmsd** | 9.159 | 2.528 | 8.471 | 10.399 | 15.113 | 5.516 | 6.835 | 7.8 | 7.417 | 11.304 |

**
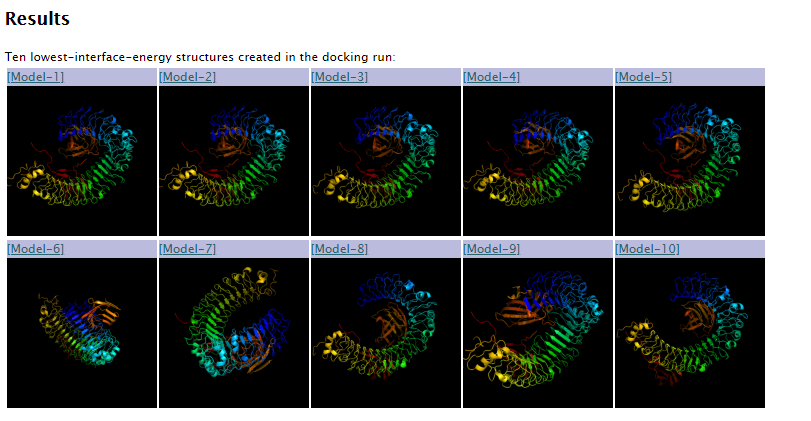
**

**Figure S5: Top 10 lowest interfacial energy of TLR-MD2 docked structure with FN-EDB by RosettaDock**

**
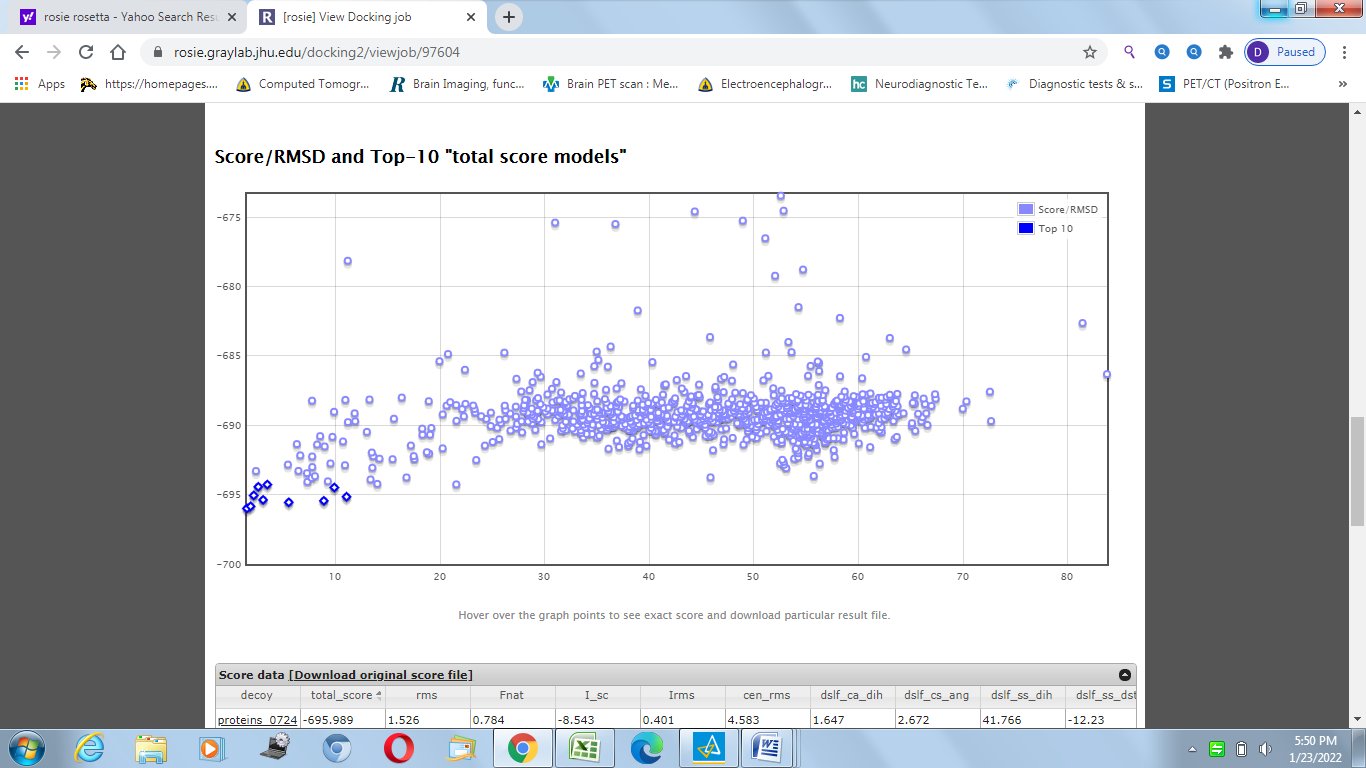
**

**Figure S6: The score v/s RMSD plot of 1000 docked mTLR4-MD2-FN-EDB models generated by RosettaDock, from which top 10 low scoring models are highlighted in dark blue**

**Identifying interfacial residues**

The tables below describe the interfacial residues present between TLR4 and FN-EDA/B in each model along with their defined atom distances (Table S4 for mTLR4-FN-EDA; S5 for hTLR4-FN-EDA; and S6 for mTLR4-FN-EDB)

**Table S4: Proximal residual atom distances for mouse TLR4-FN-EDA top model complex using UCSF Chimera**

| **S. No.** | **mTLR4** | | **mFN-EDA** | | **Distance (Å)** |
| --- | --- | --- | --- | --- | --- |
|  | **Residue** | **Atom** | **Residue** | **Atom** |  |
| 1 | ILE35 | 1HG2 | LYS6 | 3HZ | 3.308 |
| 2 | ASN204 | 1HD2 | GLU67 | OE1 | 3.666 |
| 3 | HIS228 | HE1 | PRO39 | 2HG | 2.768 |
| 4 | ARG256 | 1HH2 | PRO39 | 2HB | 2.241 |
| 5 | ASP285 | 2HB | GLU40 | 1HB | 3.543 |
| 6 | SER 309 | 2HB | GLU 40 | 2HG | 1.974 |
| 7 | GLN 331 | 1HE2 | GLU 40 | 2HG | 2.509 |
| 8 | LYS 352 | 2HZ | ARG 63 | 1HH2 | 2.98 |
| 9 | SER 353 | HG | ILE 43 | 2HG2 | 4.302 |
| 10 | THR 355 | HG1 | ILE 43 | 3HG2 | 4.125 |
| 11 | SER 374 | HG | ARG 44 | 2HH2 | 2.618 |
| 12 | TYR 375 | HE1 | ILE 43 | 3HG2 | 4.877 |
| 13 | TYR 375 | 1HB | ARG 44 | 2HH1 | 2.788 |
| 14 | TYR 375 | HE1 | GLU 45 | 2HG | 2.66 |
| 15 | ARG 400 | 1HH1 | ARG 44 | 1HH1 | 4.177 |
| 16 | HIS 401 | HE2 | GLU 45 | OE1 | 1.697 |
| 17 | HIS 424 | HE1 | LEU 46 | 1HD1 | 4.214 |
| 18 | HIS 424 | HE1 | PHE 47 | HD1 | 3.079 |
| 20 | TYR 449 | 1HB | PHE 47 | 1HB | 2.937 |
| 21 | LYS 475 | 3HZ | ARG 31 | 2HH1 | 3.627 |
| 22 | THR 497 | HG1 | ALA 49 | 3HB | 3.149 |
| 23 | PHE 498 | HE1 | SER 30 | 1HB | 2.388 |
| 24 | GLN 521 | 1HE2 | ASP 51 | OD2 | 3.179 |
| 25 | LEU 522 | 1HD1 | SER 30 | 2HB | 2.453 |
| 26 | ASN 524 | 2HD2 | ASP 77 | OD1 | 3.492 |
| 27 | PHE 570 | HZ | GLN 28 | 2HG | 3.53 |

**Table S5: Proximal residual atom distances for human TLR4-FN-EDA top model complex using UCSF Chimera**

| **S. No.** | **hTLR4** | | **hFN-EDA** | | **Distance (Å)** |
| --- | --- | --- | --- | --- | --- |
|  | **Residue** | **Atom** | **Residue** | **Atom** |  |
| 1 | VAL32 | 3HG1 | ALA94 | 1HB | 2.319 |
| 2 | HIS256 | HE2 | GLU53 | OE2 | 4.380 |
| 3 | GLU287 | OE2 | GLU53 | OE2 | 3.688 |
| 4 | ARG355 | 1HH1 | ASP51 | OD2 | 2.086 |
| 5 | PHE377 | HE1 | ALA49 | O | 4.889 |
| 6 | LYS402 | 3HZ | SER30 | 1HB | 4.429 |
| 7 | TYR403 | 1HB | ALA49 | 3HB | 4.509 |
| 8 | GLU425 | OE1 | ARG31 | 2HD | 2.519 |
| 9 | HIS426 | 1HB | PHE47 | HB2 | 2.051 |
| 10 | ILE450 | 2HG1 | ARG31 | 2HH1 | 2.181 |
| 11 | TYR451 | 2HB | PHE47 | HE2 | 3.800 |
| 12 | GLU474 | OE1 | ARG33 | 2HH2 | 1.772 |
| 13 | VAL475 | 3HG2 | GLU45 | 2HB | 4.878 |
| 14 | LYS477 | 1HZ | GLU45 | O | 4.764 |
| 15 | LYS477 | 1HZ | LEU46 | 2HD1 | 4.994 |
| 16 | THR499 | OG1 | GLU45 | OE1 | 2.718 |
| 17 | PHE500 | 1HB | GLU45 | OE1 | 2.579 |
| 18 | PHE500 | HE1 | HIS44 | ND1 | 2.849 |
| 19 | VAL524 | 1HG2 | ILE43 | O | 2.783 |
| 20 | ASN526 | 2HD2 | HIS44 | HE1 | 4.333 |
| 21 | VAL548 | 1HG2 | ILE43 | H | 2.194 |
| 22 | PHE573 | HE2 | GLU40 | N | 2.716 |
| 23 | GLU578 | 1HE2 | ARG61 | 2HH2 | 4.713 |
| 24 | VAL602 | 2HG1 | GLU40 | O | 2.728 |
| 25 | GLU603 | 1HB | GLU40 | OE2 | 2.528 |

**Table S6: Proximal residual atom distances for TLR4-FN-EDB model complex using UCSF Chimera**

| **S. No.** | **TLR4** | | **FN-EDB** | | **Distance (Å)** |
| --- | --- | --- | --- | --- | --- |
|  | **Residue** | **Atom** | **Residue** | **Atom** |  |
| 1 | HIS 429 | HD2 | ASN 30 | OD1 | 3.224 |
| 2 | TYR 454 | HD2 | ASN 30 | 2HB | 2.422 |
| 3 | ASN 456 | 1HD2 | PRO 28 | 1HB | 2.653 |
| 4 | THR 457 | 3HG2 | SER 58 | 2HB | 3.513 |
| 5 | LYS 458 | 2HD | ASP 56 | OD1 | 2.737 |
| 6 | ASN 479 | OD1 | ILE 34 | 1HG2 | 4.750 |
| 7 | SER 480 | HG | ILE 34 | HB | 2.314 |
| 8 | GLN 505 | OE1 | ASN 82 | 2HD2 | 2.612 |
| 9 | HIS 527 | HE1 | GLU 85 | 1HG | 3.955 |
| 10 | ARG 553 | 2HH2 | GLU 85 | OE2 | 1.876 |

**Docking validation in RosettaDock**

The top pose of the docking complex obtained both for FN-EDA and FN-EDB were used for docking validation through re-docking, shown below (Figure S7- mouse TLR4-MD2-FN-EDA; Figure S8- human TLR4-MD2-FN-EDA; and Figure S9- TLR4-MD2-FN-EDB)


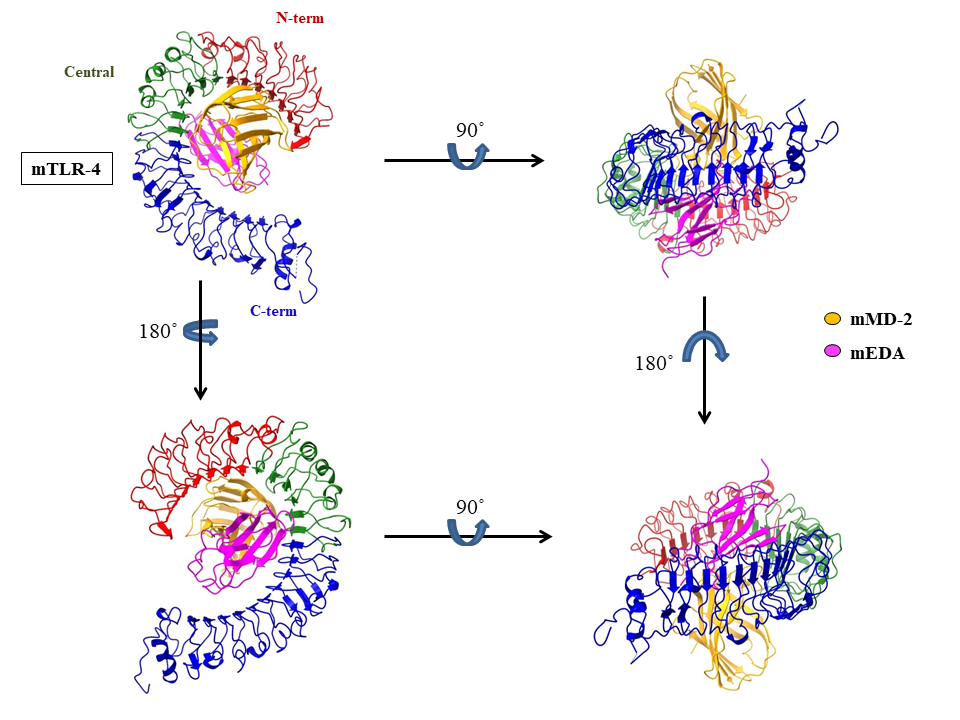


**Figure S7: Input model of mouse TLR4-MD2-FN-EDA for docking validation**


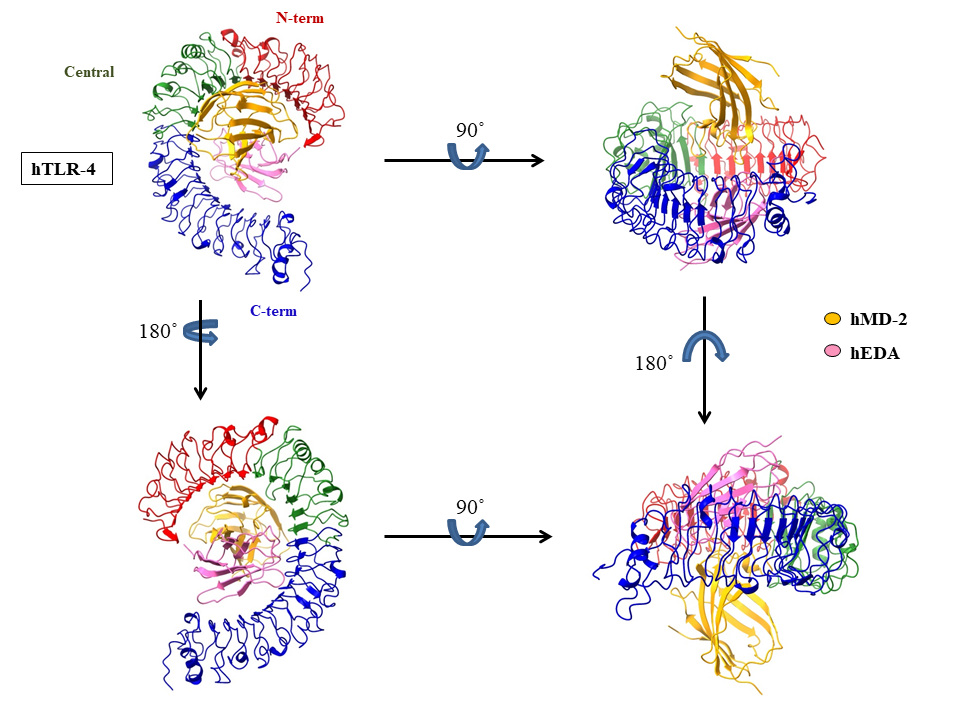


**Figure S8: Input model of human TLR4-MD2-FN-EDA for docking validation**


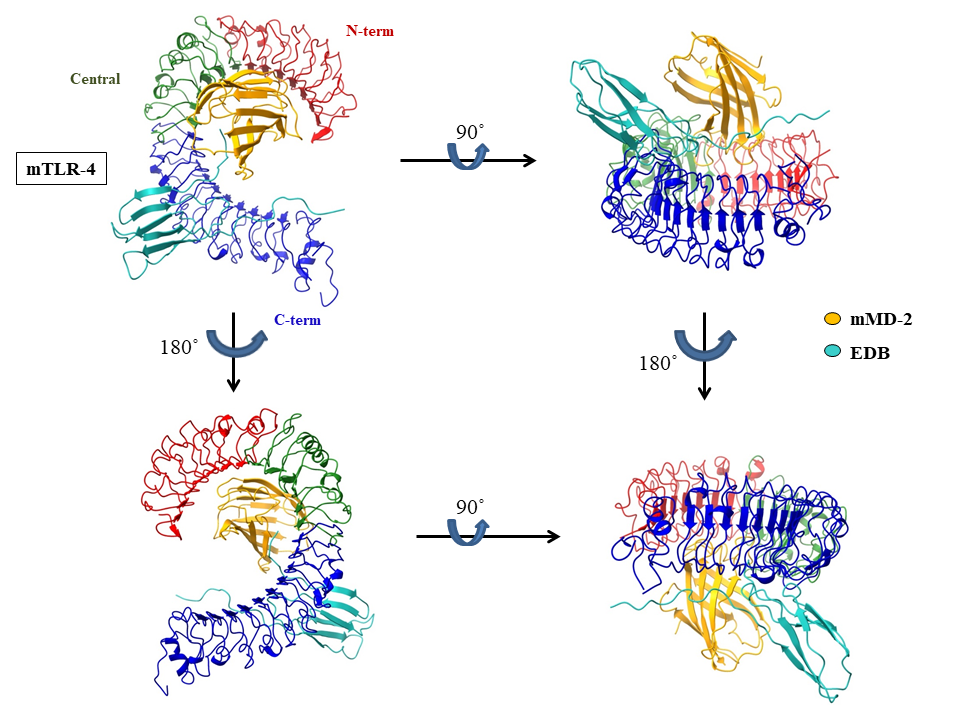


**Figure S9: Input model TLR4-MD2-FN-EDB for docking validation**

**Table S7: RosettaDock validation output of mouse TLR4-FN-EDA complex docked with MD2**

| **decoy** | **proteins_0023** | **proteins_0123** | **proteins_0069** | **proteins_0055** | **proteins_0727** | **proteins_0871** | **proteins_0022** | **proteins_0076** | **proteins_0981** | **proteins_0031** |
| --- | --- | --- | --- | --- | --- | --- | --- | --- | --- | --- |
| **total_score** | -698.4 | -698.2 | -697.8 | -697.3 | -696.1 | -695.4 | -695.3 | -695.2 | -694.8 | -694.8 |
| **rms** | 2.978 | 1.791 | 1.093 | 2.696 | 24.802 | 14.483 | 14.333 | 12.28 | 24.308 | 16.453 |
| **Fnat** | 0.836 | 0.855 | 0.909 | 0.927 | 0 | 0 | 0.036 | 0.018 | 0 | 0 |
| **I_sc** | -9.998 | -10.34 | -9.065 | -9.786 | -7.114 | -6.435 | -6.688 | -5.787 | -5.758 | -6.073 |
| **Irms** | 0.746 | 0.486 | 0.521 | 0.562 | 14.592 | 7.001 | 7.551 | 6.808 | 13.875 | 8.57 |
| **cen_rms** | 4.046 | 3.929 | 4.701 | 4.992 | 24.742 | 13.292 | 14.47 | 11.215 | 22.986 | 18.562 |
| **dslf_ca_dih** | 1.649 | 1.649 | 1.649 | 1.649 | 1.649 | 1.649 | 1.649 | 1.649 | 1.649 | 1.649 |
| **dslf_cs_ang** | 2.654 | 2.654 | 2.654 | 2.654 | 2.654 | 2.654 | 2.654 | 2.654 | 2.654 | 2.654 |
| **dslf_ss_dih** | 41.779 | 41.779 | 41.779 | 41.779 | 41.779 | 41.779 | 41.779 | 41.779 | 41.779 | 41.779 |
| **dslf_ss_dst** | -12.12 | -12.12 | -12.12 | -12.12 | -12.12 | -12.12 | -12.12 | -12.12 | -12.12 | -12.12 |
| **fa_atr** | -1257 | -1255 | -1254 | -1256 | -1251 | -1252 | -1252 | -1250 | -1252 | -1248 |
| **fa_dun** | 60.438 | 60.585 | 60.354 | 60.434 | 60.511 | 60.336 | 60.587 | 60.531 | 60.509 | 60.208 |
| **fa_elec** | -8.87 | -8.816 | -8.812 | -8.875 | -8.609 | -8.395 | -8.608 | -8.57 | -8.594 | -8.547 |
| **fa_pair** | -22.01 | -21.02 | -21.15 | -21.09 | -20.59 | -20.57 | -20.93 | -20.28 | -20.01 | -20.96 |
| **fa_rep** | 57.031 | 56.62 | 56.314 | 56.47 | 56.027 | 55.762 | 56.827 | 55.58 | 56.393 | 55.624 |
| **fa_sol** | 522.22 | 519.91 | 519.74 | 521.18 | 517.12 | 518.37 | 518.66 | 516.95 | 518.25 | 516.78 |
| **hbond_bb_sc** | -14.19 | -14.44 | -14.54 | -14.58 | -13.97 | -13.94 | -14.01 | -14 | -14 | -14.04 |
| **hbond_lr_bb** | -45.67 | -45.82 | -45.67 | -45.83 | -45.67 | -45.67 | -45.67 | -45.67 | -45.67 | -45.67 |
| **hbond_sc** | -8.549 | -8.568 | -8.563 | -8.031 | -8.31 | -8.027 | -8.47 | -8.168 | -7.764 | -8.506 |
| **hbond_sr_bb** | -15.42 | -15.42 | -15.42 | -15.42 | -15.42 | -15.42 | -15.42 | -15.42 | -15.42 | -15.42 |
| **interchain_contact** | -20 | -20 | -20 | -20 | -16 | -20 | -20 | -20 | -10 | -20 |
| **interchain_env** | -80.73 | -81.08 | -80.78 | -81.19 | -77.87 | -81.34 | -83.41 | -82.02 | -78.44 | -82.96 |
| **interchain_pair** | -2.403 | -1.848 | -2.143 | -2.802 | 2.437 | 0.239 | -2.55 | 1.228 | -0.34 | 0.704 |
| **interchain_vdw** | 0.027 | 0 | 0 | 0 | 0.128 | 0 | 0.009 | 0.813 | 0.036 | 0 |
| **st_rmsd** | 4.469 | 6.323 | 8.853 | 5.954 | 24.806 | 13.728 | 7.975 | 9.726 | 22.592 | 16.247 |

**Table S8: RosettaDock validation output of human TLR4-FN-EDA complex docked with MD2**

| **decoy** | **proteins_0106** | **proteins_0798** | **proteins_0685** | **proteins_0410** | **proteins_0147** | **proteins_0028** | **proteins_0914** | **proteins_0607** | **proteins_0144** | **proteins_0338** |
| --- | --- | --- | --- | --- | --- | --- | --- | --- | --- | --- |
| **total_score** | -746.9 | -746.4 | -746 | -745.8 | -745.8 | -745.4 | -744.9 | -744.7 | -744.6 | -744.6 |
| **rms** | 0.961 | 3.456 | 2.74 | 1.923 | 3.577 | 3.498 | 8.635 | 17.931 | 5.585 | 4.806 |
| **Fnat** | 0.852 | 0.803 | 0.82 | 0.738 | 0.672 | 0.656 | 0.508 | 0.033 | 0.59 | 0.59 |
| **I_sc** | -9.452 | -9.878 | -9.999 | -9.022 | -9.084 | -8.363 | -7.124 | -6.041 | -7.31 | -7.663 |
| **Irms** | 0.432 | 0.681 | 0.609 | 0.65 | 0.915 | 1.182 | 2.007 | 9.295 | 1.358 | 1.081 |
| **cen_rms** | 3.695 | 3.219 | 4.976 | 2.022 | 5.701 | 6.261 | 4.963 | 18.254 | 7.769 | 5.029 |
| **dslf_ca_dih** | 0.607 | 0.607 | 0.607 | 0.607 | 0.607 | 0.607 | 0.607 | 0.607 | 0.607 | 0.607 |
| **dslf_cs_ang** | 2.951 | 2.951 | 2.951 | 2.951 | 2.951 | 2.951 | 2.951 | 2.951 | 2.951 | 2.951 |
| **dslf_ss_dih** | 3.802 | 3.802 | 3.802 | 3.802 | 3.802 | 3.802 | 3.802 | 3.802 | 3.802 | 3.802 |
| **dslf_ss_dst** | -13.87 | -13.87 | -13.87 | -13.87 | -13.87 | -13.87 | -13.87 | -13.87 | -13.87 | -13.87 |
| **fa_atr** | -1243 | -1243 | -1244 | -1242 | -1244 | -1243 | -1240 | -1245 | -1243 | -1243 |
| **fa_dun** | 60.995 | 61.104 | 61.025 | 60.956 | 61.011 | 60.845 | 61.25 | 61.203 | 60.929 | 60.994 |
| **fa_elec** | -8.633 | -8.699 | -8.743 | -8.507 | -8.72 | -8.639 | -8.658 | -8.324 | -8.624 | -8.755 |
| **fa_pair** | -21.76 | -22.09 | -21.33 | -21.4 | -21.55 | -21.35 | -23.05 | -21.34 | -21.39 | -22.03 |
| **fa_rep** | 44.195 | 44.438 | 44.406 | 44.33 | 44.419 | 44.462 | 44.642 | 44.551 | 44.726 | 44.545 |
| **fa_sol** | 508.9 | 509.37 | 509.91 | 507.44 | 510.58 | 509.96 | 508.09 | 511.19 | 510.14 | 510.91 |
| **hbond_bb_sc** | -14.73 | -14.75 | -15.1 | -14.78 | -14.97 | -14.89 | -14.54 | -14.69 | -14.83 | -14.72 |
| **hbond_lr_bb** | -43.62 | -43.53 | -43.57 | -43.59 | -43.6 | -43.58 | -43.44 | -43.44 | -43.44 | -43.44 |
| **hbond_sc** | -10.52 | -10.35 | -10.5 | -10.14 | -10.54 | -10.83 | -10.71 | -9.855 | -10.51 | -10.93 |
| **hbond_sr_bb** | -12.03 | -12.03 | -12.03 | -12.03 | -12.03 | -12.03 | -12.03 | -12.03 | -12.03 | -12.03 |
| **interchain_contact** | -20 | -20 | -18 | -20 | -20 | -20 | -20 | -20 | -20 | -20 |
| **interchain_env** | -86.11 | -84.39 | -84.08 | -88.14 | -86.19 | -87.17 | -84.86 | -85.66 | -86.76 | -86.83 |
| **interchain_pair** | 0.156 | -2.357 | -0.251 | -0.713 | -1.291 | -2.121 | -2.543 | 0.074 | -2.596 | -2.202 |
| **interchain_vdw** | 0 | 0.452 | 0.66 | 0.026 | 0 | 0 | 0 | 0.124 | 0.088 | 0 |
| **st_rmsd** | 4.608 | 4.159 | 7.329 | 1.651 | 7.605 | 4.473 | 3.703 | 18.868 | 8.084 | 5.002 |

**Table S9: RosettaDock validation output of TLR4-FN-EDB complex docked with MD2**

| **decoy** | **proteins_0724** | **proteins_0268** | **proteins_0711** | **proteins_0131** | **proteins_0334** | **proteins_0329** | **proteins_0654** | **proteins_0186** | **proteins_0817** | **proteins_0475** |
| --- | --- | --- | --- | --- | --- | --- | --- | --- | --- | --- |
| **total_score** | -695.989 | -695.842 | -695.552 | -695.443 | -695.377 | -695.151 | -695.056 | -694.482 | -694.434 | -694.273 |
| **rms** | 1.526 | 1.874 | 5.522 | 8.875 | 3.067 | 11.044 | 2.174 | 9.877 | 2.604 | 3.469 |
| **Fnat** | 0.784 | 0.863 | 0.49 | 0.451 | 0.627 | 0.235 | 0.725 | 0.314 | 0.843 | 0.804 |
| **I_sc** | -8.543 | -8.951 | -8.551 | -7.374 | -8.024 | -7.966 | -7.54 | -7.951 | -9.607 | -7.226 |
| **Irms** | 0.401 | 0.433 | 1.462 | 2.27 | 1.073 | 3.102 | 0.611 | 2.683 | 0.688 | 0.85 |
| **cen_rms** | 4.583 | 4.789 | 3.268 | 8.067 | 5.549 | 11.415 | 4.003 | 9.383 | 5.94 | 3.293 |
| **dslf_ca_dih** | 1.647 | 1.647 | 1.647 | 1.647 | 1.647 | 1.647 | 1.647 | 1.647 | 1.647 | 1.647 |
| **dslf_cs_ang** | 2.672 | 2.672 | 2.672 | 2.672 | 2.672 | 2.672 | 2.672 | 2.672 | 2.672 | 2.672 |
| **dslf_ss_dih** | 41.766 | 41.766 | 41.766 | 41.766 | 41.766 | 41.766 | 41.766 | 41.766 | 41.766 | 41.766 |
| **dslf_ss_dst** | -12.23 | -12.23 | -12.23 | -12.23 | -12.23 | -12.23 | -12.23 | -12.23 | -12.23 | -12.23 |
| **fa_atr** | -1262.192 | -1260.917 | -1262.221 | -1262.377 | -1260.559 | -1264.571 | -1261.447 | -1261.086 | -1264.039 | -1258.698 |
| **fa_dun** | 61.223 | 61.142 | 61.214 | 61.204 | 61.094 | 60.835 | 61.172 | 60.724 | 61.299 | 61.036 |
| **fa_elec** | -8.984 | -8.964 | -8.97 | -9.022 | -8.925 | -8.993 | -9.032 | -9.057 | -8.979 | -8.964 |
| **fa_pair** | -21.906 | -21.942 | -21.343 | -21.99 | -21.564 | -20.731 | -21.652 | -21.275 | -21.96 | -21.44 |
| **fa_rep** | 62.037 | 62.491 | 62.743 | 62.228 | 62.153 | 63.273 | 62.2 | 63.023 | 63.767 | 61.903 |
| **fa_sol** | 525.595 | 524.127 | 525.015 | 525.824 | 523.98 | 526.32 | 525.646 | 524.644 | 527.141 | 523.634 |
| **hbond_bb_sc** | -15.787 | -15.822 | -15.784 | -15.356 | -15.356 | -15.352 | -15.779 | -15.37 | -15.461 | -15.791 |
| **hbond_lr_bb** | -45.515 | -45.515 | -45.515 | -45.515 | -45.515 | -45.515 | -45.515 | -45.515 | -45.515 | -45.515 |
| **hbond_sc** | -8.804 | -8.784 | -9.034 | -8.783 | -9.027 | -8.76 | -8.994 | -8.914 | -9.032 | -8.783 |
| **hbond_sr_bb** | -15.51 | -15.51 | -15.51 | -15.51 | -15.51 | -15.51 | -15.51 | -15.51 | -15.51 | -15.51 |

RMSD matrix for mouse TLR4-MD2-FN-EDA, human TLR4-MD2-FN-EDA and TLR4-MD2-FN-EDB are given below from which the selected models are highlighted in green, which fall under low RMSD region.

Model 1, 2, 3, 4 from mouse TLR4-MD2-FN-EDA (Figure S10); Model 1, 2, 4, 7, 8 and 9 from human TLR4-MD2-FN-EDA (Figure S11); and Model 1, 2, 3, 4 and 5 from mouse TLR4-MD2-FN-EDB (Figure S12) were considered.


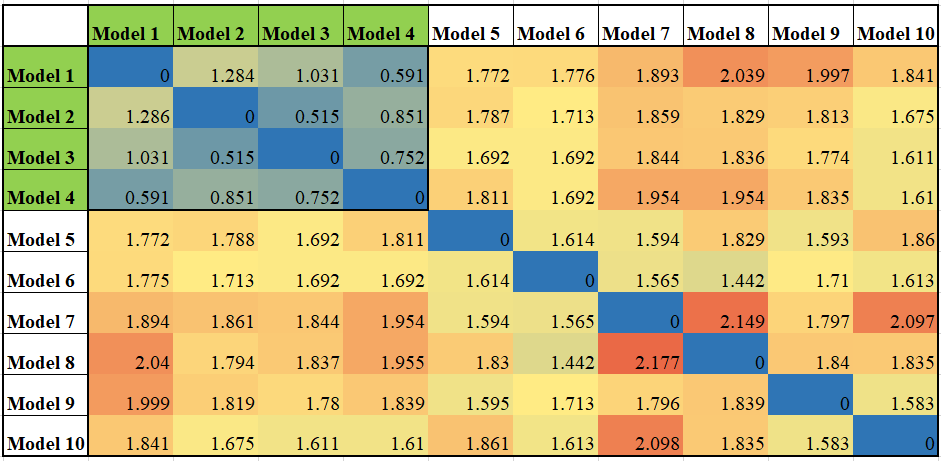


**Figure S10: RMSD matrix for mouse TLR4-MD2-FN-EDA (selected models highlighted in green)**


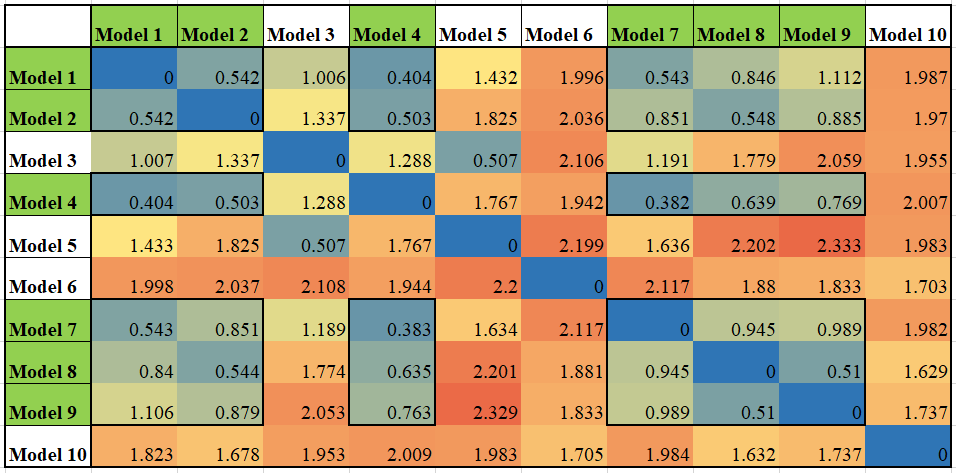


**Figure S11: RMSD matrix for human TLR4-MD2-FN-EDA (selected models highlighted in green)**


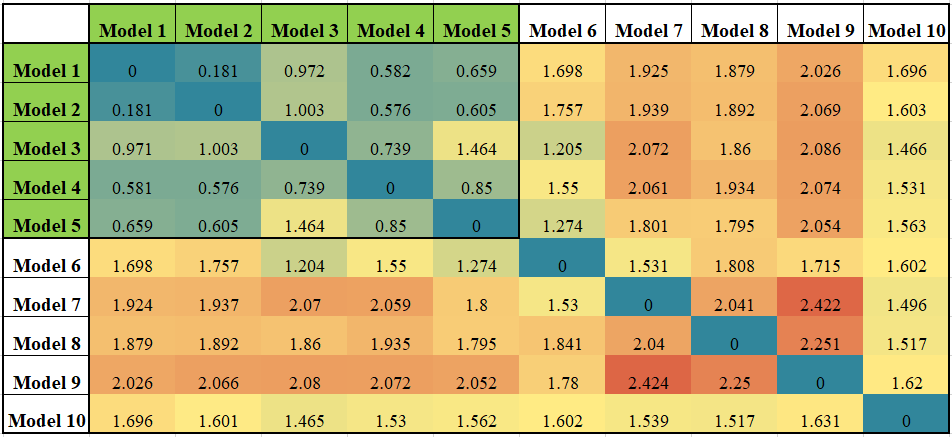


**Figure S12: RMSD matrix for mouse TLR4-MD2-FN-EDB (selected models highlighted in green)**

**Prime Energies**

Below are the prime energies and PIPER scores of top 3 models obtained for human TLR4-MD2-FN-EDA and TLR4-MD2-FN-EDB complexes (Table S10) from the low RMSD filtered models from the RMSD matrix. Also, the individual prime energies of human TLR4, MD2 and FN-EDA (Table S11), and mouse TLR4, MD2 and FN-EDB (Table S12) are given below.

**Table S10: Prime energies and PIPER scores of low RMSD models of human TLR4-MD2-FN-EDA and mouse TLR4-MD2-FN-EDB complexes**

| **Model Name** | **Model no.** | **Prime Energy (kcal/mol)** | **PIPER pose score** |
| --- | --- | --- | --- |
| Human TLR4-MD2-FN-EDA | Model 1 | -36126.78 | -601.002 |
|  | Model 2 | -36116.78 | -587.884 |
|  | Model 4 | -36115.5 | -575.250 |
| Mouse TLR4-MD2-FN-EDB | Model 2 | -30866.0 | -221.554 |
|  | Model 3 | -30677.1 | -235.892 |
|  | Model 5 | -30401.8 | -232.791 |

**Table S11: Individual prime energies of the binding proteins along with their total prime energy in human**

| **Proteins** | **Prime Energy (kcal/mol)** |
| --- | --- |
| Human TLR4 | -26598.56 |
| Human MD2 | -5687.67 |
| Human EDA | -3586.93 |
| **Total Prime Energy =** | -35873.16 |

**Table S12: Individual prime energies of the binding proteins along with their total prime energy in mouse FN-EDB model**

| **Proteins** | **Prime Energy (kcal/mol)** |
| --- | --- |
| Mouse TLR4 | -26131.9 |
| Mouse MD2 | -5454.3 |
| Mouse EDB | -4210.6 |
| **Total Prime Energy =** | -35796.8 |

**FN-EDA filtered docked model outputs of Ramachandran plot in PROCHECK: -**


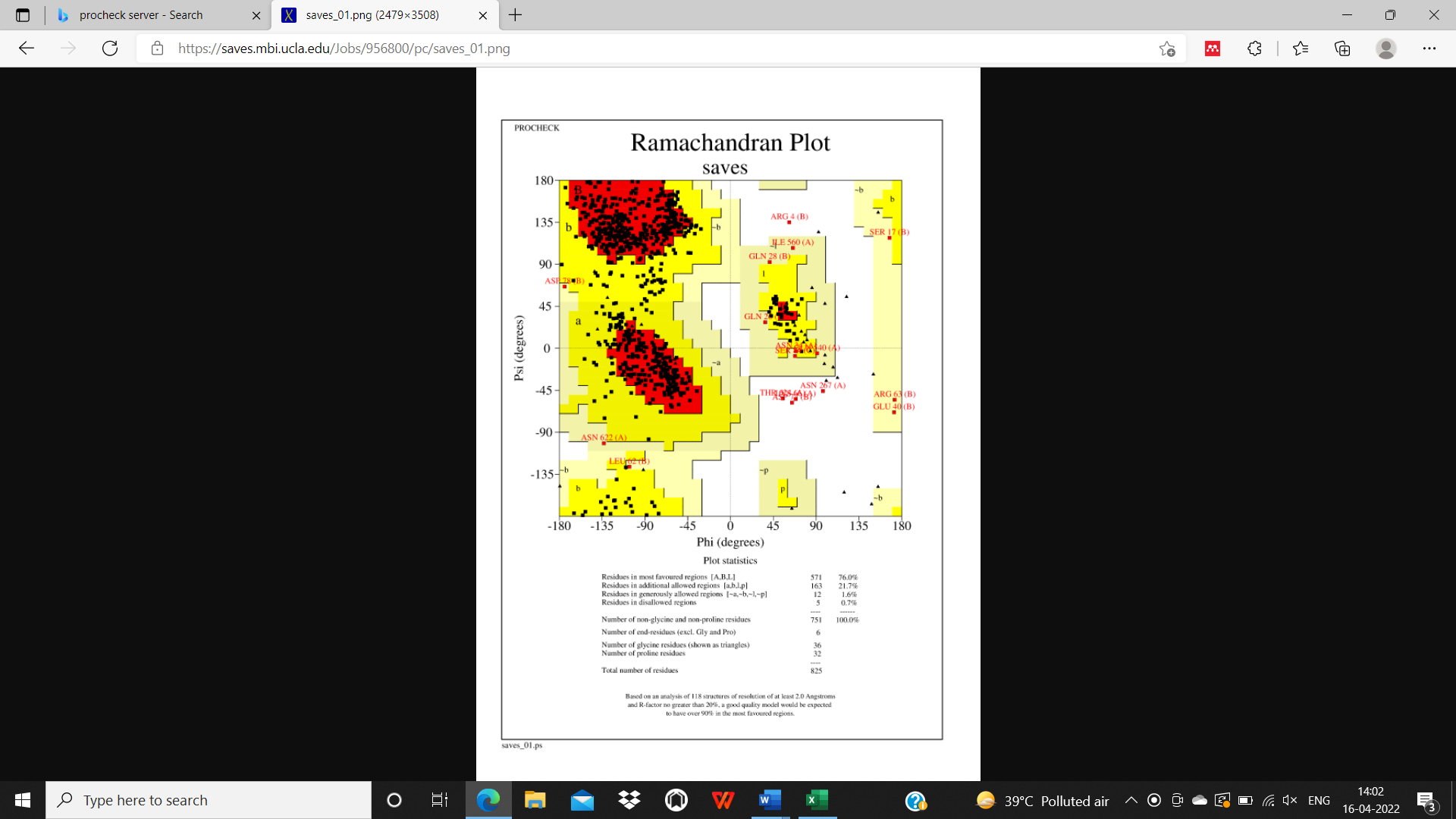


1. **Mouse TLR4-MD2-FN-EDA model**


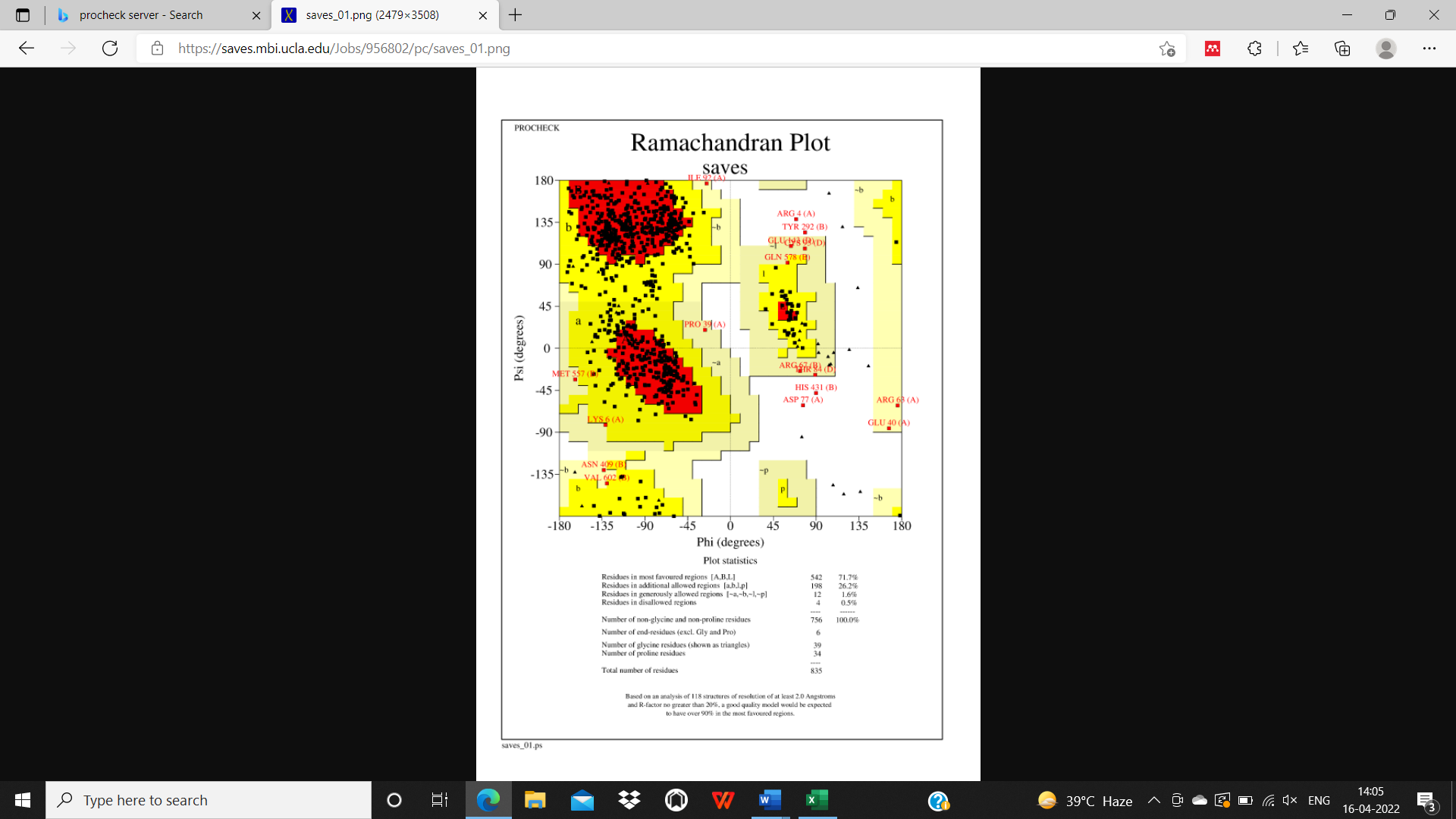


1. **Human TLR4-MD2-FN-EDA Model**

**
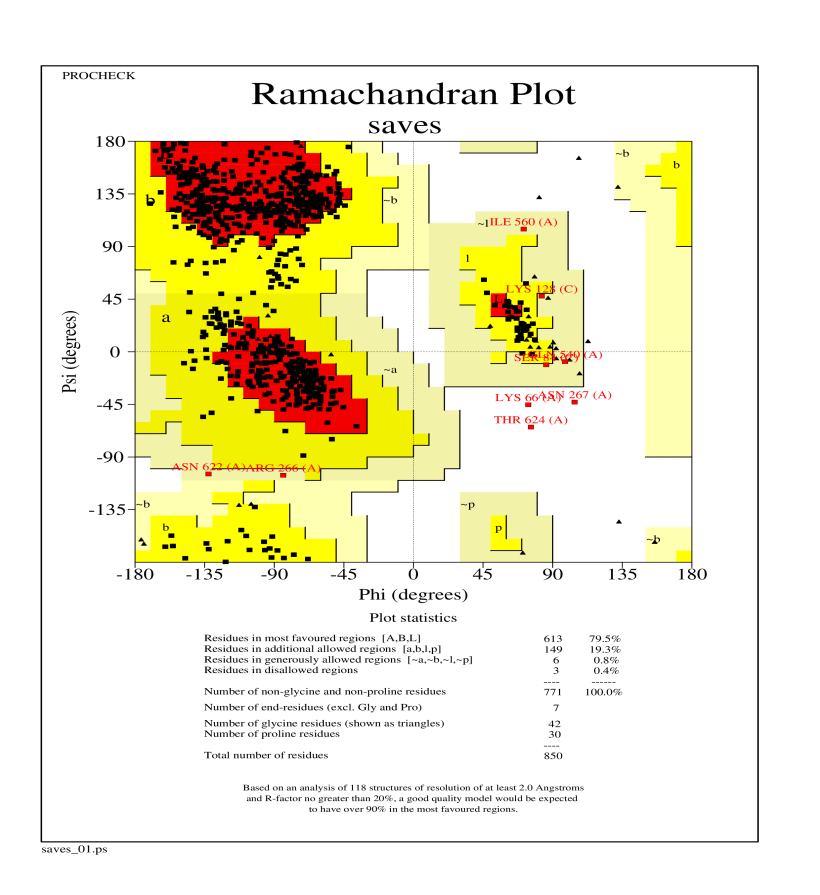
**

1. **Mouse TLR4-MD2-FN-EDB Model**

**Figure S13: Ramachandran plots in PROCHECK; a) mouse TLR4-MD2-FN-EDA; b) human TLR4-MD2-FN-EDA; and c) mouse TLR4-MD2-FN-EDB**

**SLiM sequence**

- **Mouse FN-EDA**


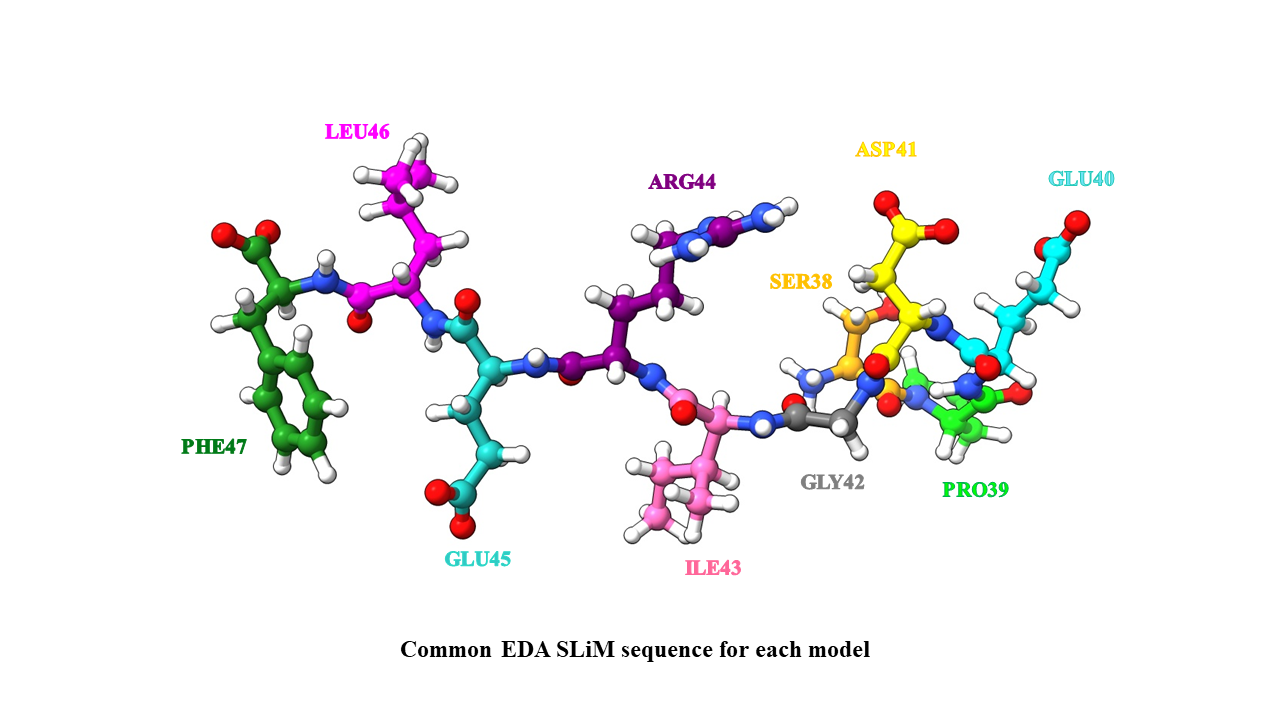


**Figure S14: Representation of the common FN-EDA SLiM sequence obtained for all TLR4-MD2-FN-EDA mouse models**


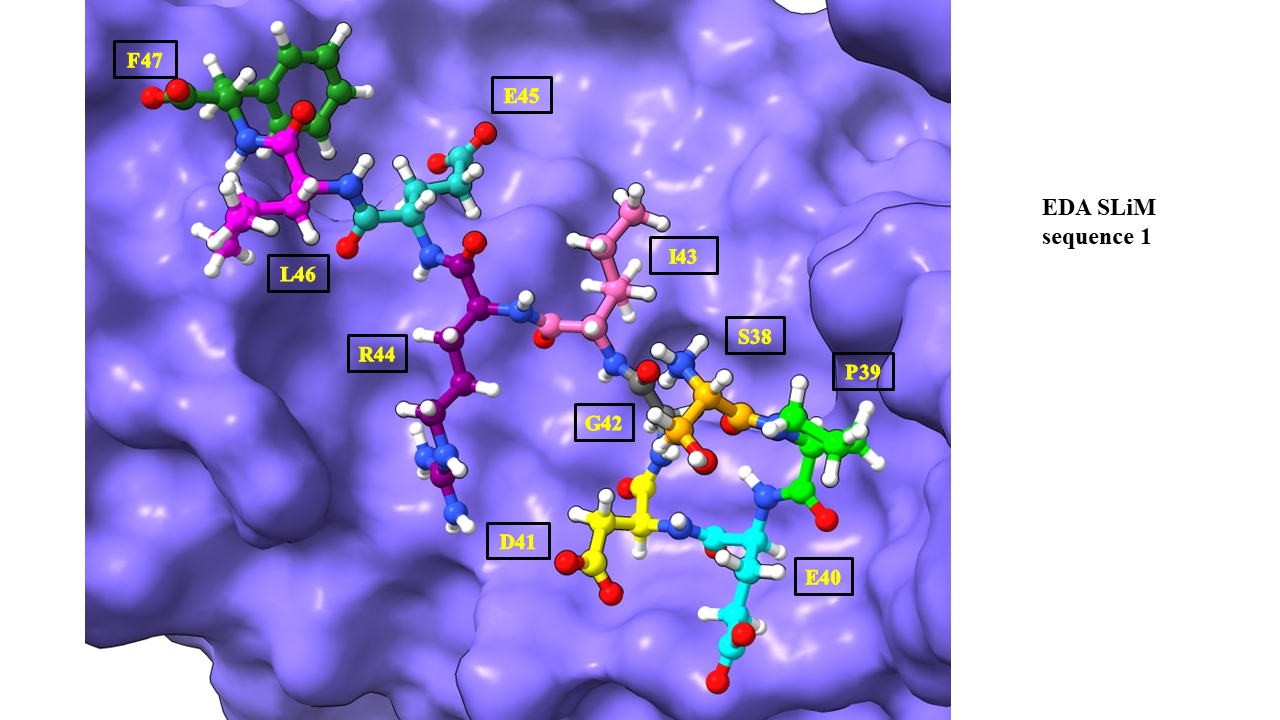


**Figure S15: Depiction of common mouse FN-EDA slim sequence “SPEDGIRELF” (ball and stick structure) on the TLR4 receptor surface (purple surface)**


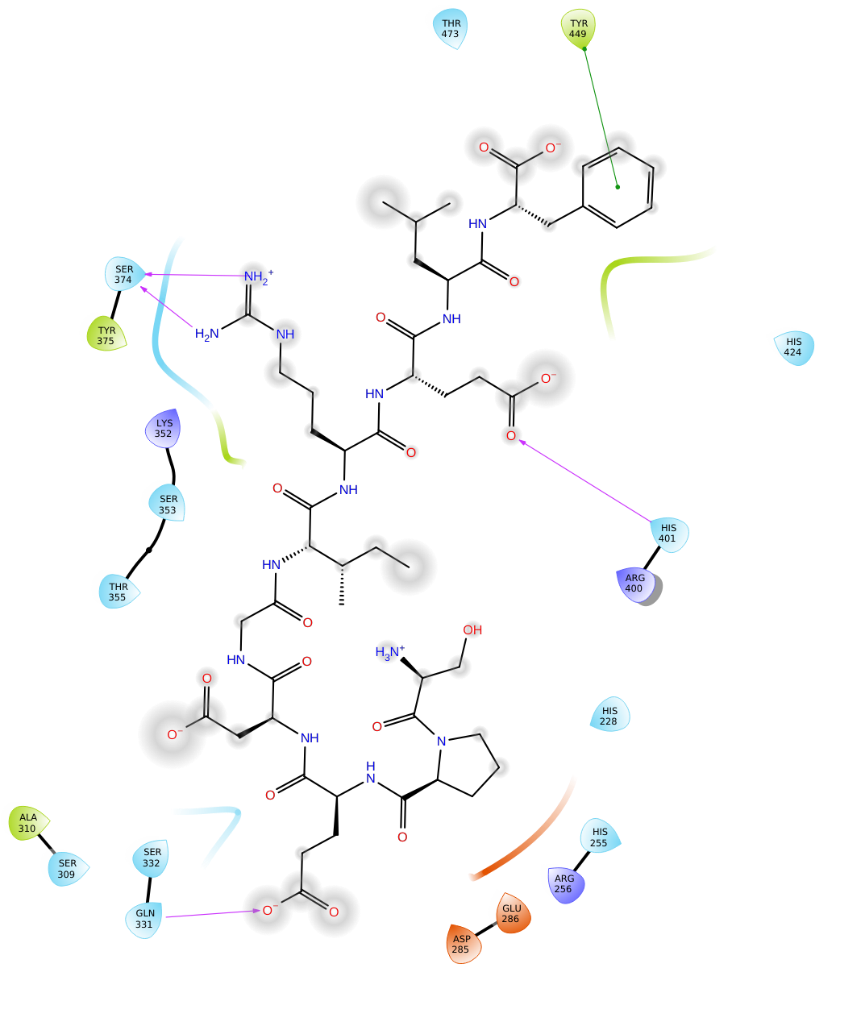


**Figure S16: 2D view ligand interaction diagram of mouse FN-EDA SLiM sequence with TLR4**

- **Human FN-EDA**


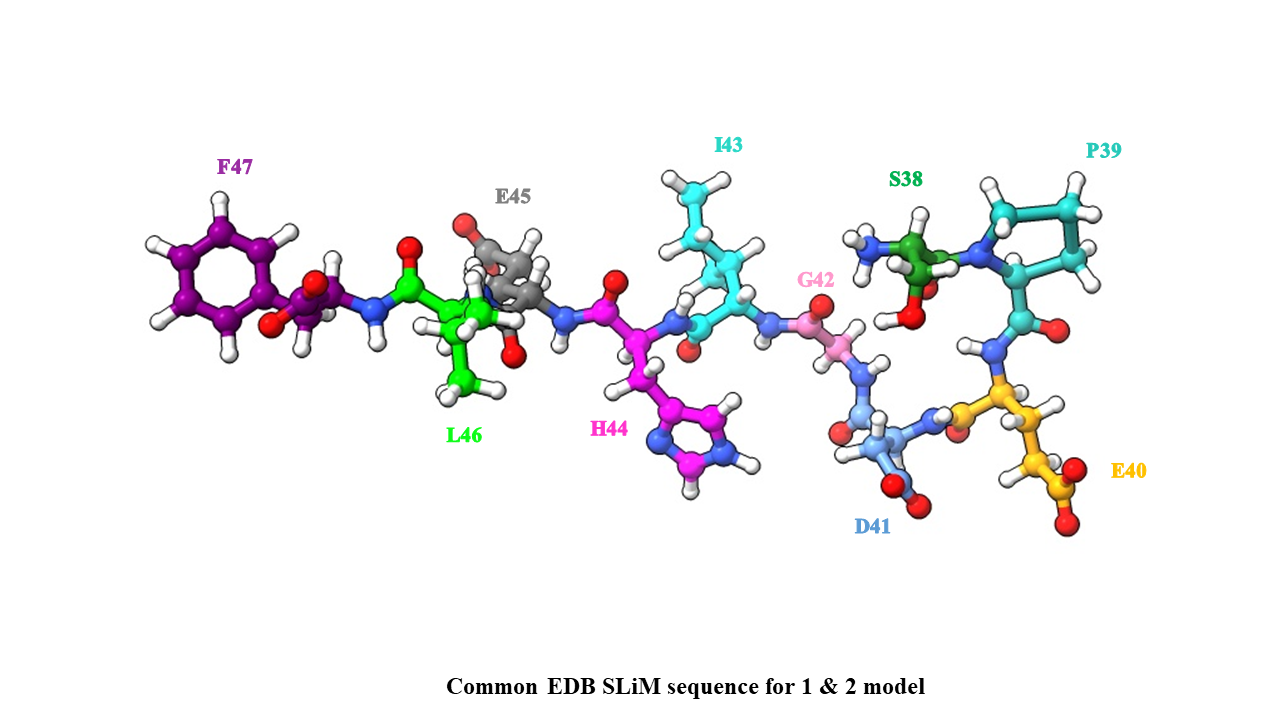


**Figure S17: Representation of the FN-EDA SLiM sequence obtained for the TLR4-MD2-FN-EDA human top model**


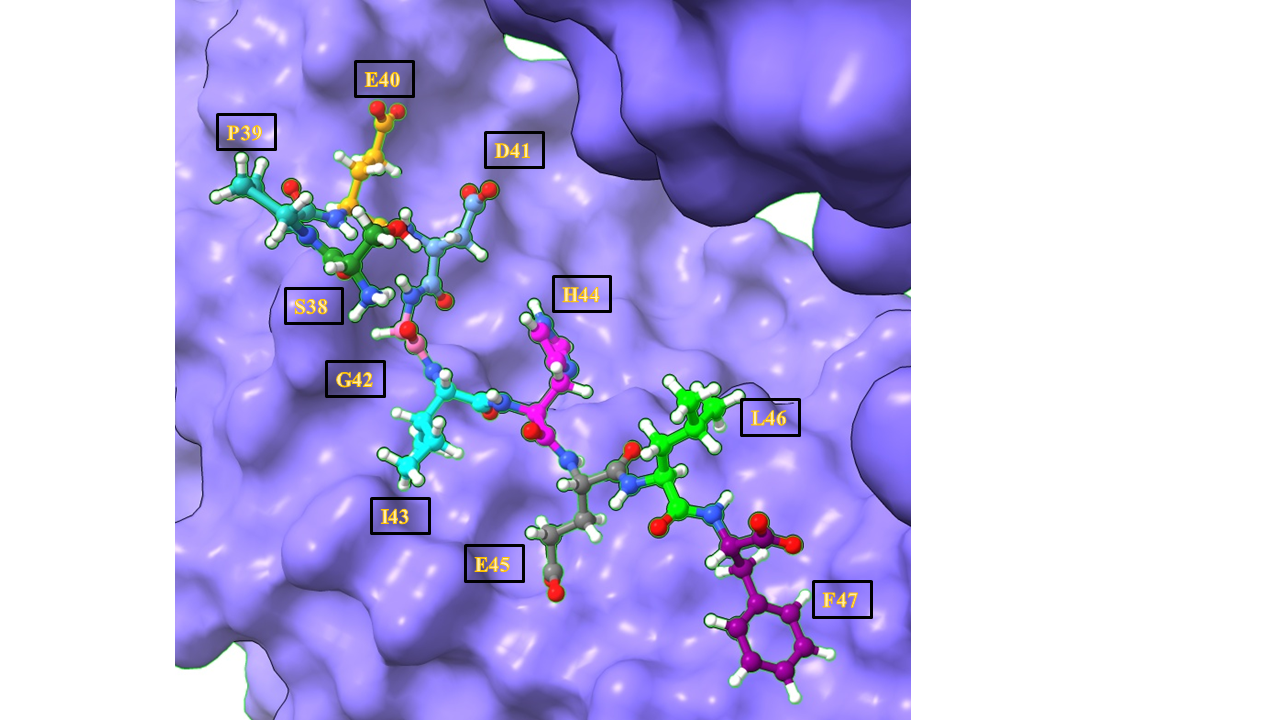


**Figure S18: Depiction of common human FN-EDA slim sequence “SPEDGIHELF” (ball and stick structure) on the TLR4 receptor surface (purple surface)**

**Table S13: SLiM sequences (small linear interacting motif) of human FN-EDA identified by Rosetta server (Peptiderive) for filtered TLR4-MD2-FN-EDA docked model complexes**

| **Model**  **Name** | **Model No.** | **Receptor Chain**  **(hTLR4)** | **Partner Chain**  **(hFN-EDA)** | **Peptide**  **Sequence Position**  **(Residue no.)** | **Best identified linear Peptide Sequence**  **(SLiM sequence)** | **Interface Score**  **(kcal /mol)** | **Total interface score**  **(REU)** | **Relative Interface score (%)** |
| --- | --- | --- | --- | --- | --- | --- | --- | --- |
| Human TLR4-MD2-FN-EDA | 1 | B | A | 38 | SPEDGIHELF | -9.713 | -22.11 | 51.22 |
|  | 2 | B | A | 35 | TYSSPEDGIH | -6.753 | -21.96 | 48.02 |


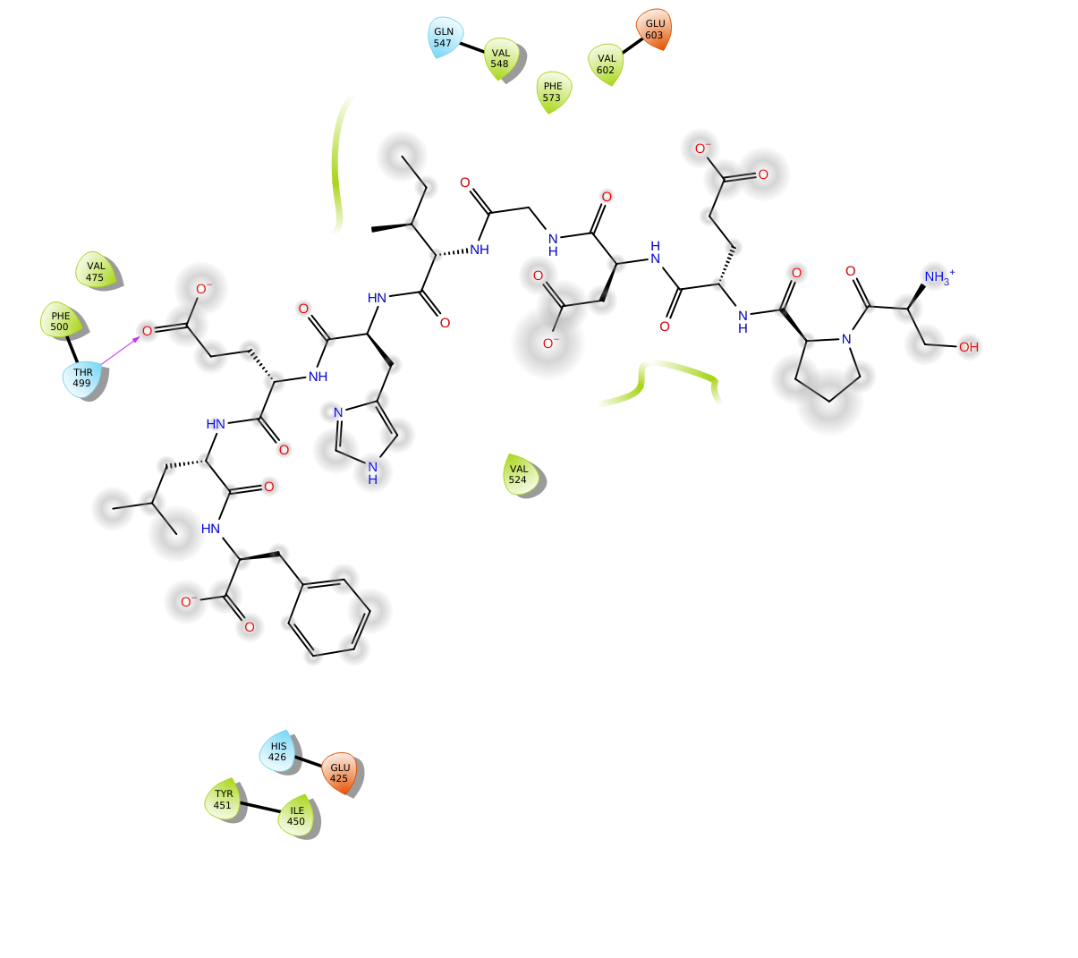


**Figure S19: 2D view ligand interaction diagram of human FN-EDA SLiM sequence 1 with TLR4**

**
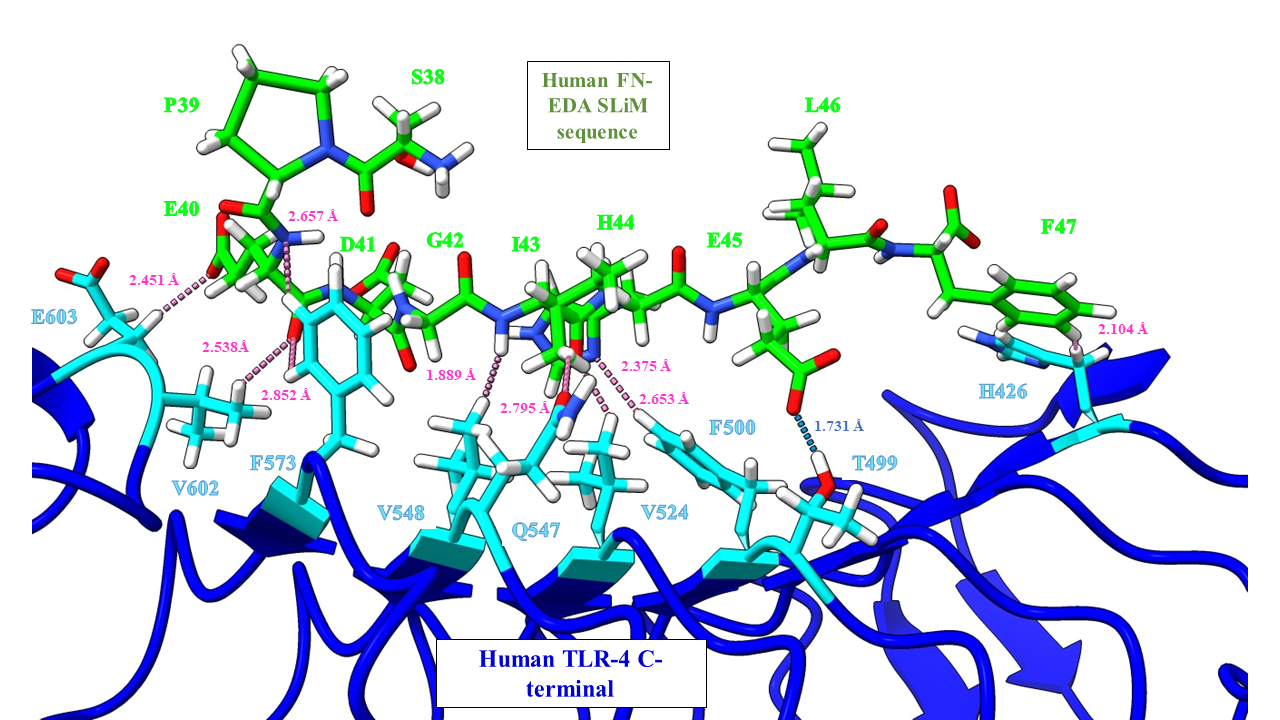
**

**Figure S20: Protein-protein interactions of human FN-EDA SLiM sequence 1 (orange sticks) with TLR4 central domain (green sticks) and C-terminal (blue sticks), identified using ChimeraX (Goddard et al., 2018) (Pettersen et al., 2021), showing hydrogen bonding (blue dashes) and Van-der-Waal interactions (pink dashes). FN-EDA interacts with the residues of central and C-terminal domain of TLR4**

- **FN-EDB**


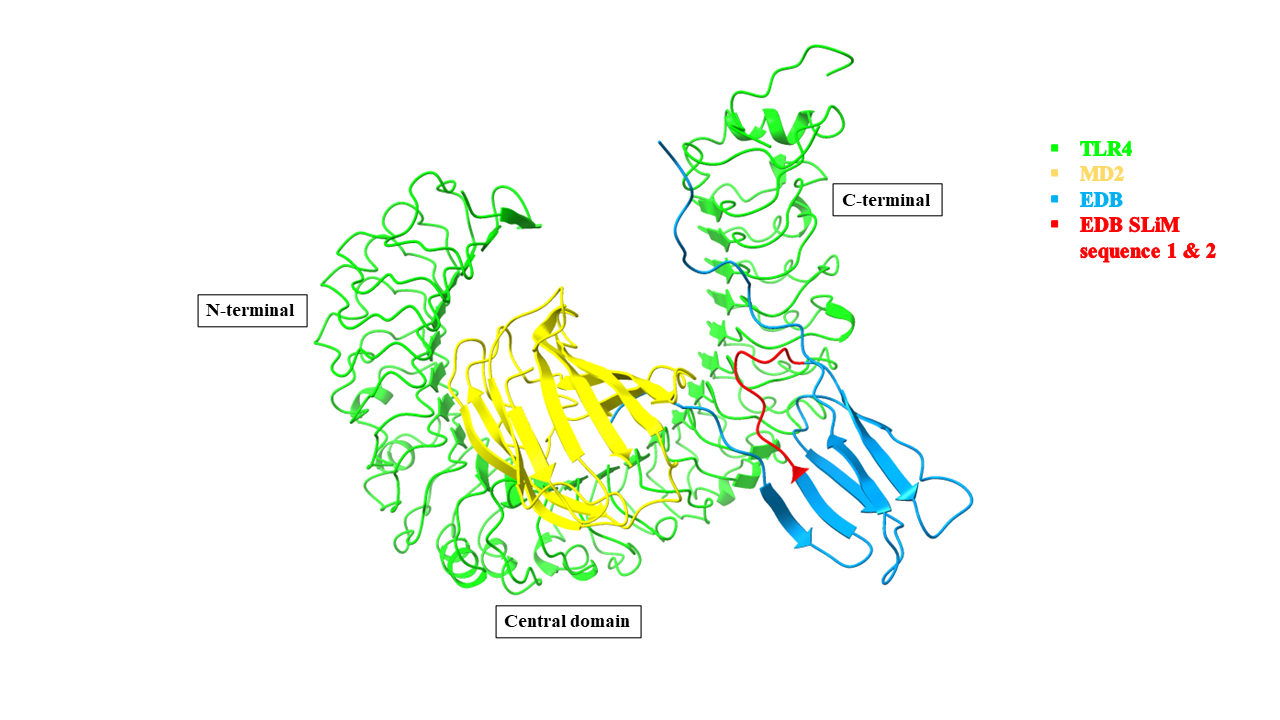


**Figure S21: Representation of TLR4-MD2-FN-EDB depicting the interacting peptide (SLiM sequence) of FN-EDB (highlighted in red) with the TLR4 in ChimeraX. FN-EDB SLiM sequence is present near the outer C-terminal region of TLR4**


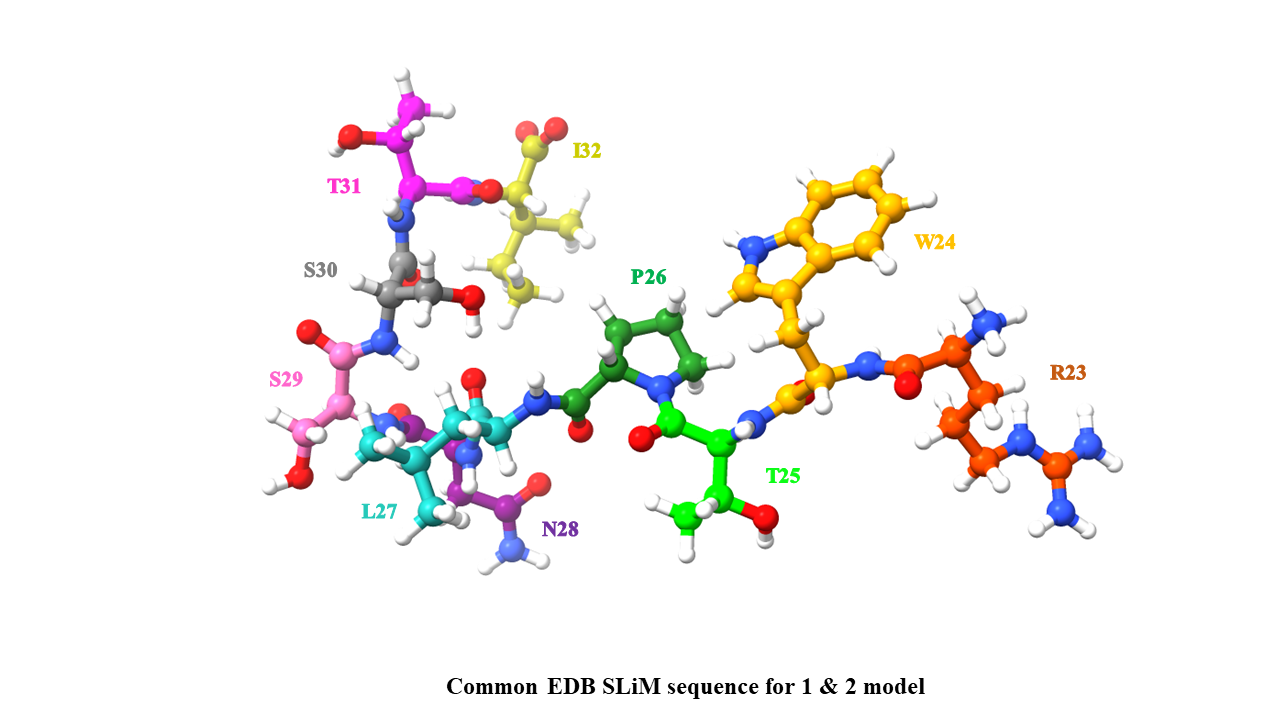


**Figure S22: Representation of FN-EDB SLiM sequence**


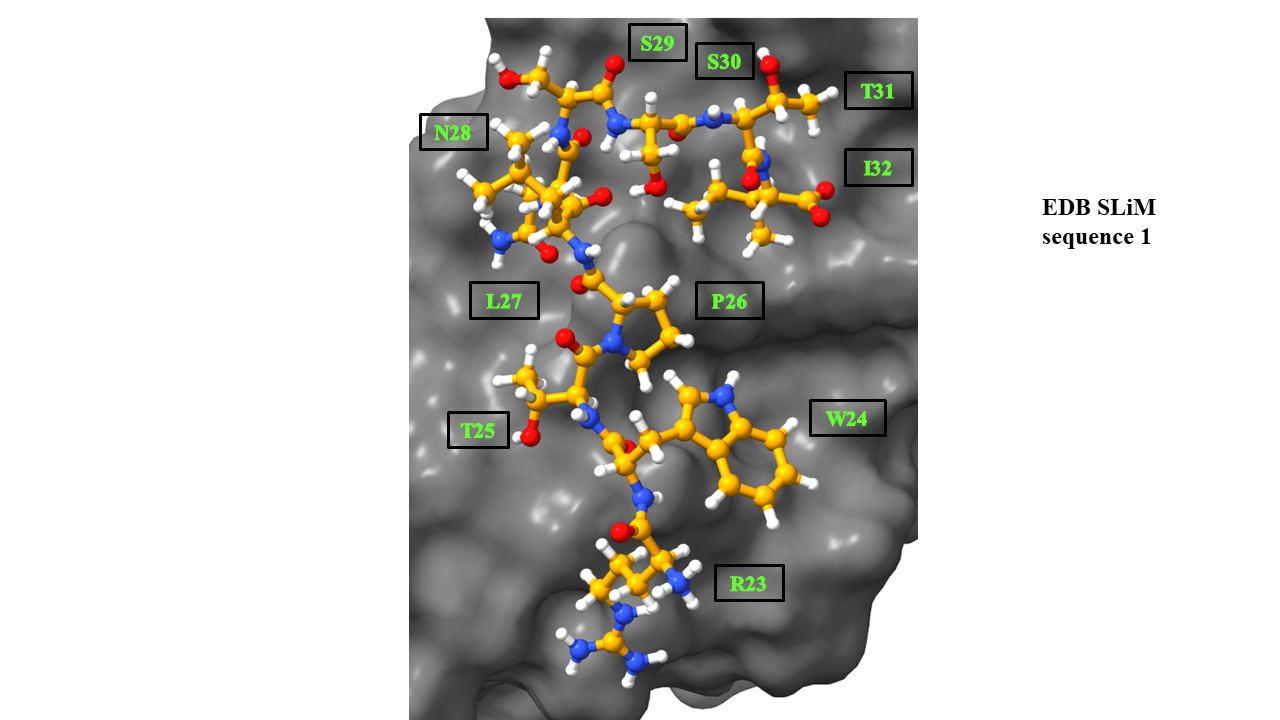


**Figure S23: Depiction of FN-EDB slim sequence (orange sticks) on the TLR4 receptor surface**

**Table S14: SLiM sequences (small linear interacting motif) of FN-EDB identified by Rosetta server (Peptiderive) for filtered FN-EDB and TLR4-MD2 docked model complexes**

| **Model Name** | **Model No.** | **Receptor Chain**  **(TLR4)** | **Partner Chain**  **(FN-EDB)** | **Peptide**  **Sequence Position**  **(Residue no.)** | **Best identified linear Peptide Sequence**  **(SLiM sequence)** | **Interface Score**  **(kcal /mol)** | **Total interface score**  **(REU)** | **Relative Interface score (%)** |
| --- | --- | --- | --- | --- | --- | --- | --- | --- |
| TLR4-MD2-FN-EDB | 1 | A | E | 23 | RWTPLNSSTI | -9.455 | -18.70 | 43.05 |
|  | 2 | A | E | 23 | RWTPLNSSTI | -9.425 | -18.46 | 42.63 |


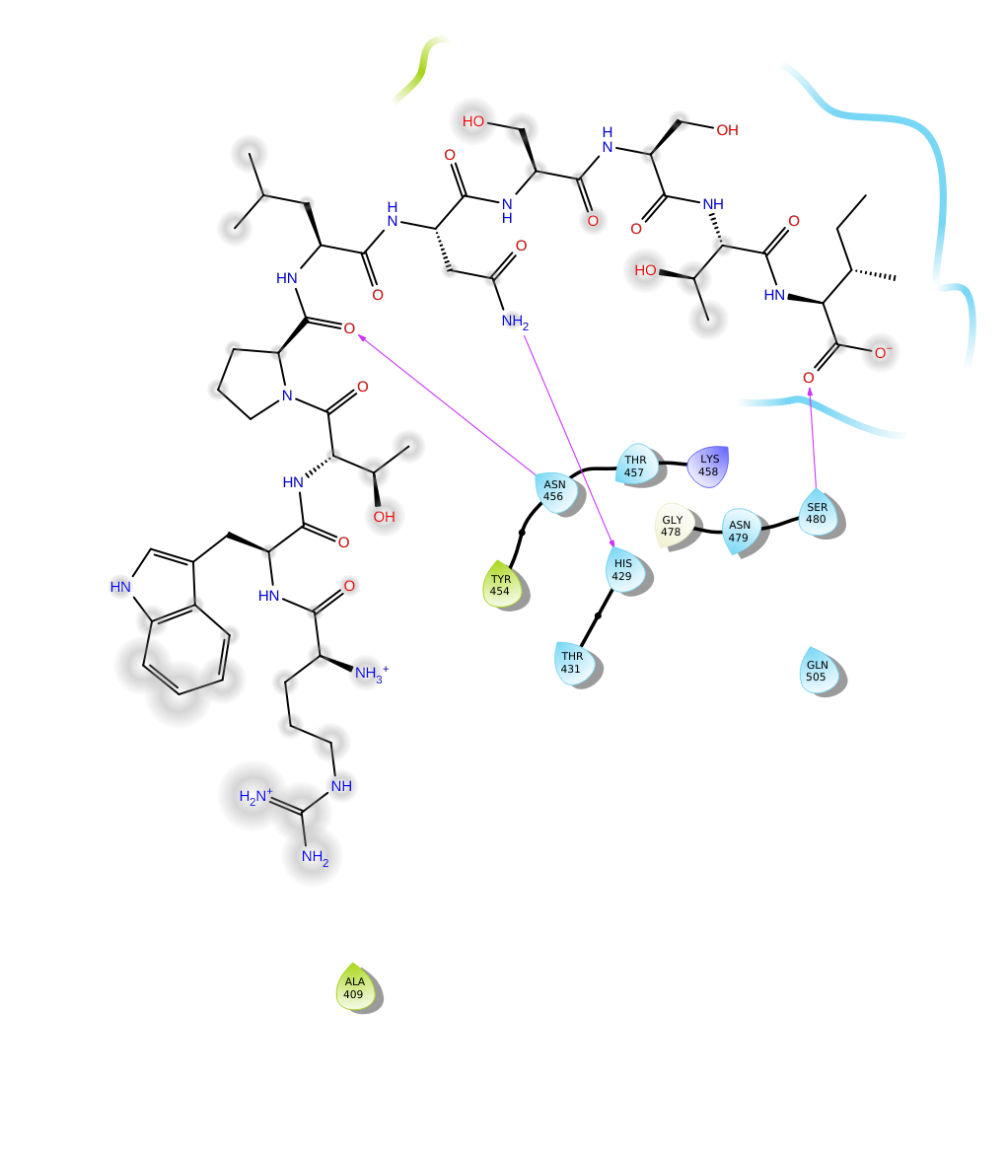


**Figure S24: 2D view ligand interaction diagram of FN-EDB SLiM sequence with TLR4**


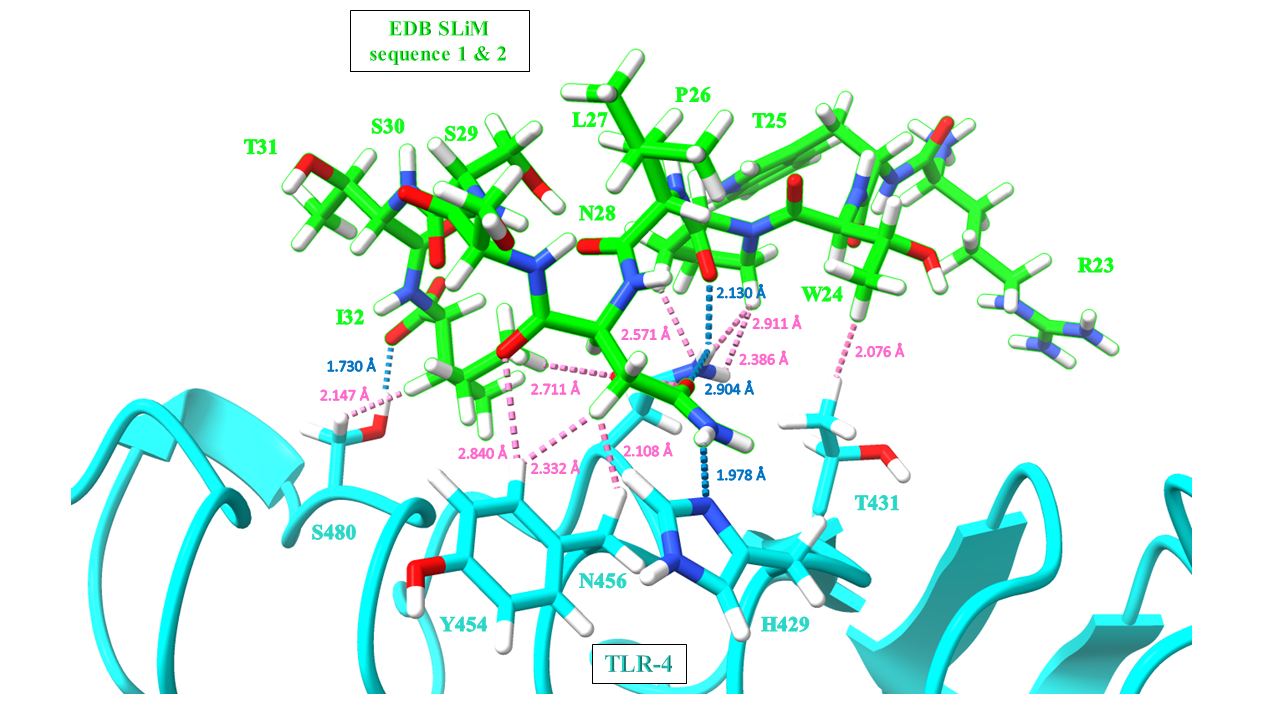


**Figure S25: Protein-protein interactions of FN-EDB common SLiM sequence (green sticks) with TLR4 residues (cyan sticks). FN-EDB here interacts with the outer C-terminal domain of TLR4.**

**Table S15: Residue scanning results after mutation showing change in stability and prime energy after every residue mutation in mouse TLR4-MD2-FN-EDA**

| **Residue before mutation** | **Residue after mutation** | **Δ Stability (solvated)** | **Δ Prime Energy (kcal/mol)** |
| --- | --- | --- | --- |
| SER-38 | ALA | 3.16 | 9.33 |
| PRO-39 | ALA | 2.03 | -27.99 |
| GLU-40 | ALA | 6.99 | 31.18 |
| ASP-41 | ALA | 8.37 | 44.48 |
| GLY-42 | ALA | 0.09 | 0.64 |
| ILE-43 | ALA | 12.57 | -4.95 |
| ARG-44 | ALA | 17.66 | 45.04 |
| GLU-45 | ALA | 6.35 | 29.9 |
| LEU-46 | ALA | 12.36 | 6.03 |
| PHE-47 | ALA | 11.02 | 13.57 |


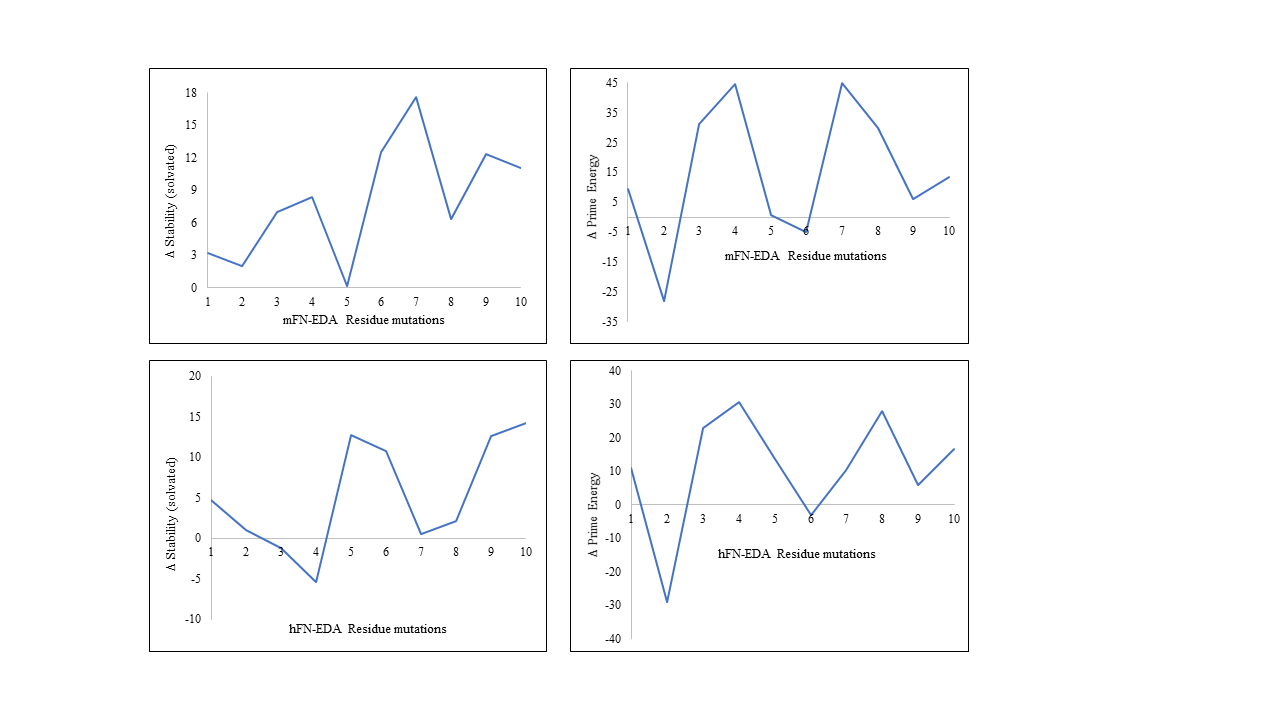


**Figure S26: Δ Stability (solvated) and Δ Prime Energy graphs depicting difference in stabilities and prime energies after mutation of each residue in SLiM sequence to Alanine through Residue scanning in mouse FN-EDA**

**Table S16: Prime energies of the human FN-EDA model complexes obtained after mutations in SLiM sequence**

| **Mutations in SLiM sequence** | **Prime Energy (kcal/mol)** |
| --- | --- |
| E40 + D41 to Alanine | -36002.8 |
| Whole SLiM sequence mutation to Alanine (“SPEDGIHELF”) | -35706.6 |

**Table S17: Residue scanning results after mutation showing change in stability and prime energy after every residue mutation in human TLR4-MD2-FN-EDA**

| **Residue before mutation** | **Residue after mutation** | **Δ Stability (solvated)** | **Δ Prime Energy (kcal/mol)** |
| --- | --- | --- | --- |
| SER-38 | ALA | 4.66 | 10.83 |
| PRO-39 | ALA | 0.95 | -29.07 |
| GLU-40 | ALA | -1.18 | 23.01 |
| ASP-41 | ALA | -5.42 | 30.69 |
| GLY-42 | ALA | 12.73 | 13.47 |
| ILE-43 | ALA | 10.8 | -3.09 |
| HIS-44 | ALA | 0.45 | 10.4 |
| GLU-45 | ALA | 2.12 | 27.83 |
| LEU-46 | ALA | 12.66 | 5.71 |
| PHE-47 | ALA | 14.2 | 16.75 |


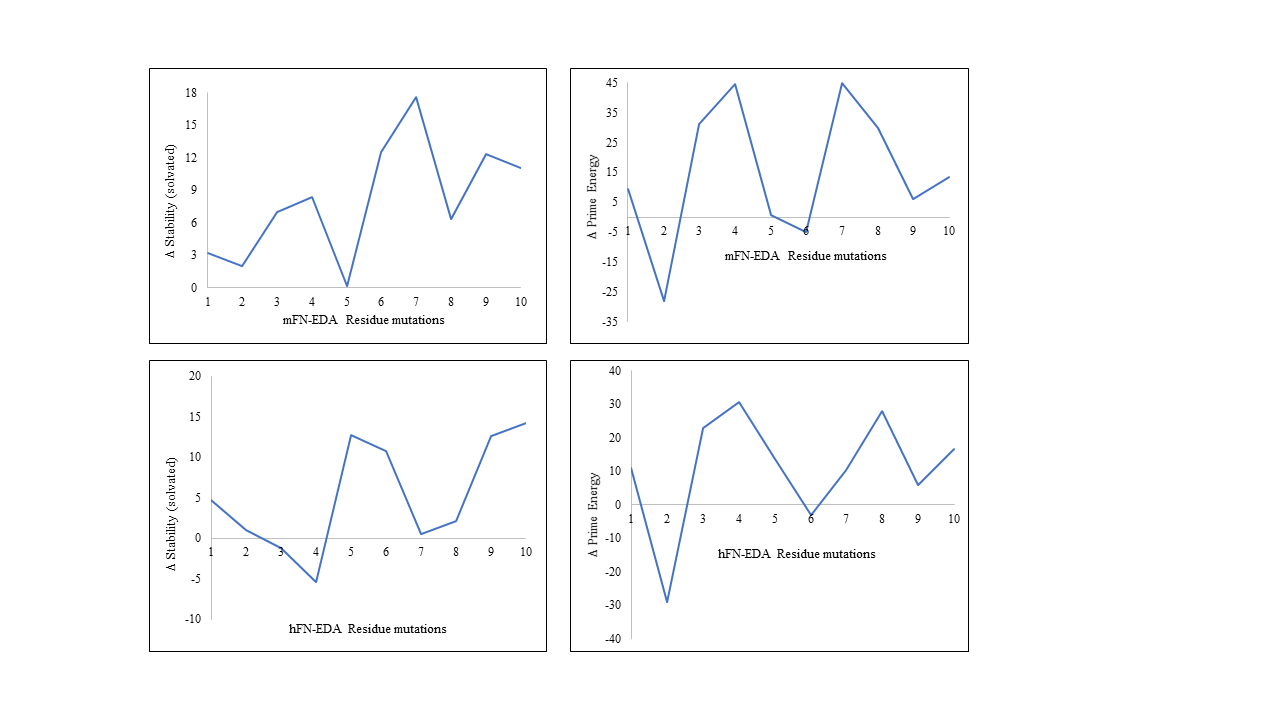
**Figure S27: Δ Stability (solvated) and Δ Prime Energy graphs depicting difference in stabilities and prime energies after mutation of each residue in SLiM sequence to Alanine through Residue scanning in human FN-EDA from TLR4-MD2-FN-EDA human model**

**Table S18: HDOCK scores of the FN-EDA joint segments with FNIII(11) and FNIII(12) docked with TLR4-MD2 in both mouse and human**

| **Model organism** | **Model Name** | **HDOCK score (kcal/mol)** |
| --- | --- | --- |
| Mouse | TLR4-MD2-FNIII(11)-EDA | -241.08 |
|  | TLR4-MD2-FNIII(11)-EDA-(12) | -271.7 |
| Human | TLR4-MD2-FNIII(11)-EDA | -246.1 |
|  | TLR4-MD2-FNIII(11)-EDA-(12) | -277.5 |

**Table S19: SLiM (Small Linear interacting Motif) sequences identified by Rosetta server (Peptiderive) for TLR4-MD2 complexes docked with FNIII (11)-EDA and FNIII (11)-EDA-(12) segments for both mouse and human**

| **Model**  **Name** | **Peptide**  **Sequence Position**  **(Residue no.)** | **Best identified linear Peptide Sequence**  **(SLiM sequence) of FN-EDA segments with TLR4** | **Interface Score**  **(kcal /mol)** | **Total interface score**  **(REU)** | **Relative Interface score (%)** |
| --- | --- | --- | --- | --- | --- |
| Mouse TLR4-MD2-FNIII (11)-EDA | 133 | SPEDGIRELF | -13.797 | -17.39 | 79.32 |
| Mouse TLR4-MD2-FNIII (11)-EDA-(12) | 199 | TPTSFTAQWI | -6.417 | -16.14 | 39.75 |
| Human TLR4-MD2-FNIII (11)-EDA | 133 | SPEDGIHELF | -17.229 | -25.66 | 67.16 |
| Human TLR4-MD2-FNIII (11)-EDA-(12) | 133 | SPEDGIHELF | -4.360 | -20.77 | 21.00 |

**Table S20: Distances between interacting residues of mouse TLR4 and FN-EDA**

| **S. No.** | **mTLR4 interacting residue (atom type)** | **mFN-EDA interacting residue (atom type)** | **Distance (Å)** |
| --- | --- | --- | --- |
| 1 | ASP285 (2HB) | PRO39 (O) | 2.816 |
| 2 | GLU286 (OE1) | PRO39 (2HB) | 2.587 |
| 3 | SER309 (2HB) | GLU40 (2HG) | 2.190 |
| 4 | GLN331 (1HB) | GLU40 (O) | 2.379 |
| 5 | SER332 (2HB) | ASP41 (O) | 2.367 |
| 6 | SER353 (2HB) | ASP41 (O) | 2.736 |
| 7 | SER374 (OG) | ARG44 (2HH1) | 2.050 |
| 8 | SER374 (OG) | ARG44 (2HH2) | 2.084 |
| 9 | TYR375 (HE1) | GLU45 (2HG) | 2.214 |
| 10 | HIS401 (HE2) | GLU45 (OE1) | 1.932 |
| 11 | HIS424 (HE1) | PHE47 (H) | 2.145 |


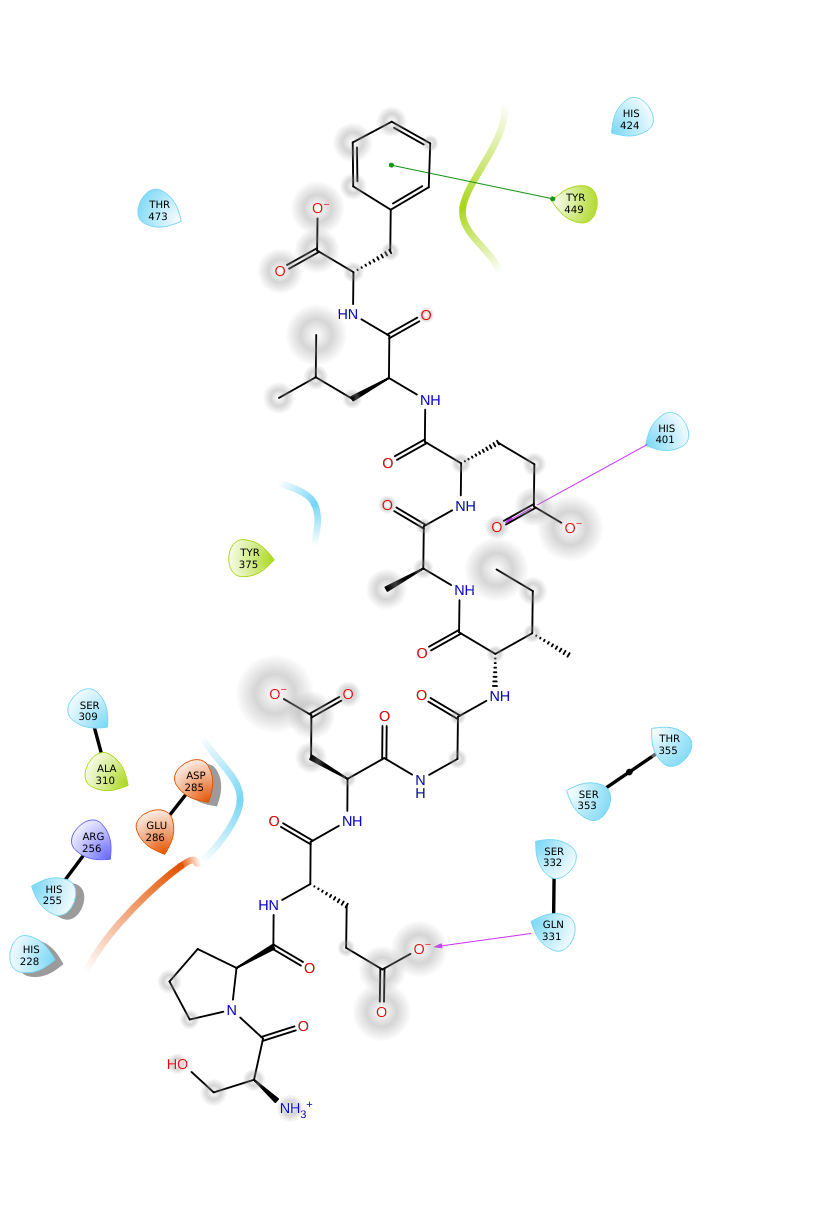


**Figure S28: 2D view ligand interaction diagram of mutated (Arg44 to Ala, according to Figure S26) mouse FN-EDA SLiM sequence with TLR4. Here, Ala44 in place of Arg44, does not seem to form H-bonds with any TLR4 residue.**

**Table S21: Conservation scores of mTLR4 interacting residues with mFN-EDA by ConSurf. Scale from 0 to 9 represents the residues as variable (0-4) to conserved (5-9) respectively. Out of the 9 interacting mTLR4 residues with mFN-EDA, 6 are variable, indicating FN-EDA selective interaction with TLR4 in mouse.**

| **S. No.** | **mTLR4 interacting residues** | **Conservation scores (Scale = 0-9)** |
| --- | --- | --- |
| 1 | ASP285 | 3 |
| 2 | GLU286 | 8 |
| 3 | SER309 | 8 |
| 4 | GLN331 | 3 |
| 5 | SER332 | 2 |
| 6 | SER353 | 4 |
| 7 | SER374 | 8 |
| 8 | TYR375 | 4 |
| 9 | HIS401 | 4 |
| 10 | HIS424 | 3 |

**Table S22: Conservation scores of hTLR4 interacting residues with hFN-EDA by ConSurf. Scale from 0 to 9 represents the residues as variable (0-4) to conserved (5-9) respectively. Out of the 9 interacting hTLR4 residues with hFN-EDA, 6 are variable, indicating FN-EDA selective interaction with TLR4 in humans.**

| **S. No.** | **hTLR4 interacting residues** | **Conservation scores (Scale = 0-9)** |
| --- | --- | --- |
| 1 | HIS426 | 3 |
| 2 | THR499 | 7 |
| 3 | PHE500 | 1 |
| 4 | VAL524 | 2 |
| 5 | GLN547 | 5 |
| 6 | VAL548 | 4 |
| 7 | PHE573 | 1 |
| 8 | VAL602 | 6 |
| 9 | GLU603 | 2 |

**Molecular Dynamics of FN-EDA models**


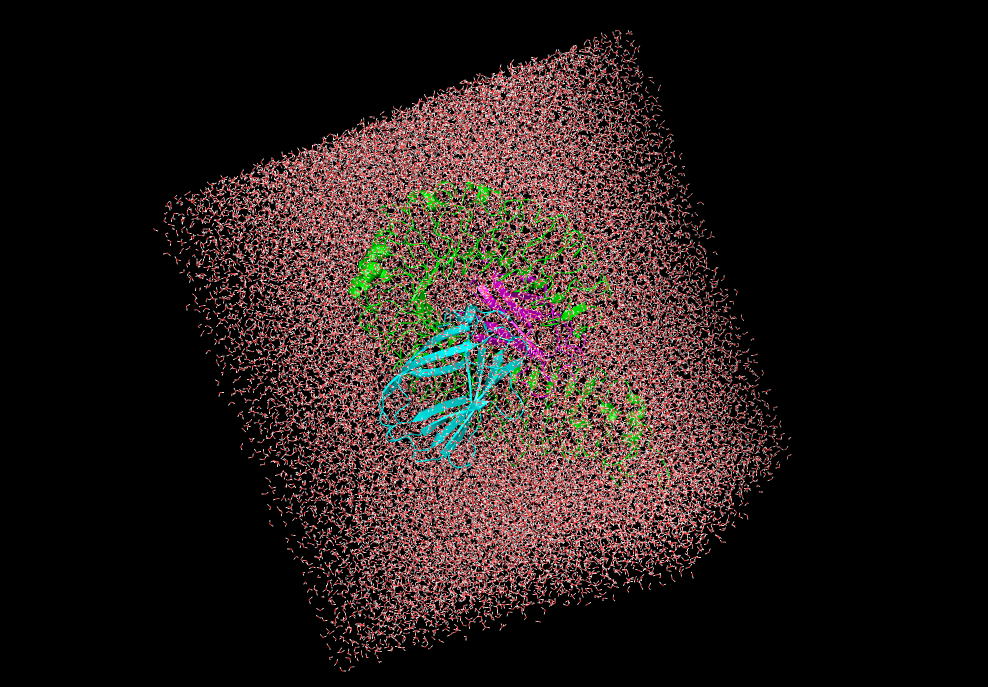


**Figure S29: TLR4-MD2-FN-EDA mouse model in an orthorhombic SPC solvent system**

**Table S23: Average potential energy of the mouse mFN-EDA model complexes during the MD simulation**

| **Model Name** | **Model no.** | **Average potential energy during simulation (kcal/mol)** |
| --- | --- | --- |
| Mouse TLR4-MD2-FN-FN-EDA | 1 | -272785.457 |
| Human TLR4-MD2-FN-EDA | 1 | -293384.802 |

- Outputs for human TLR4-MD2-FN-EDA 1


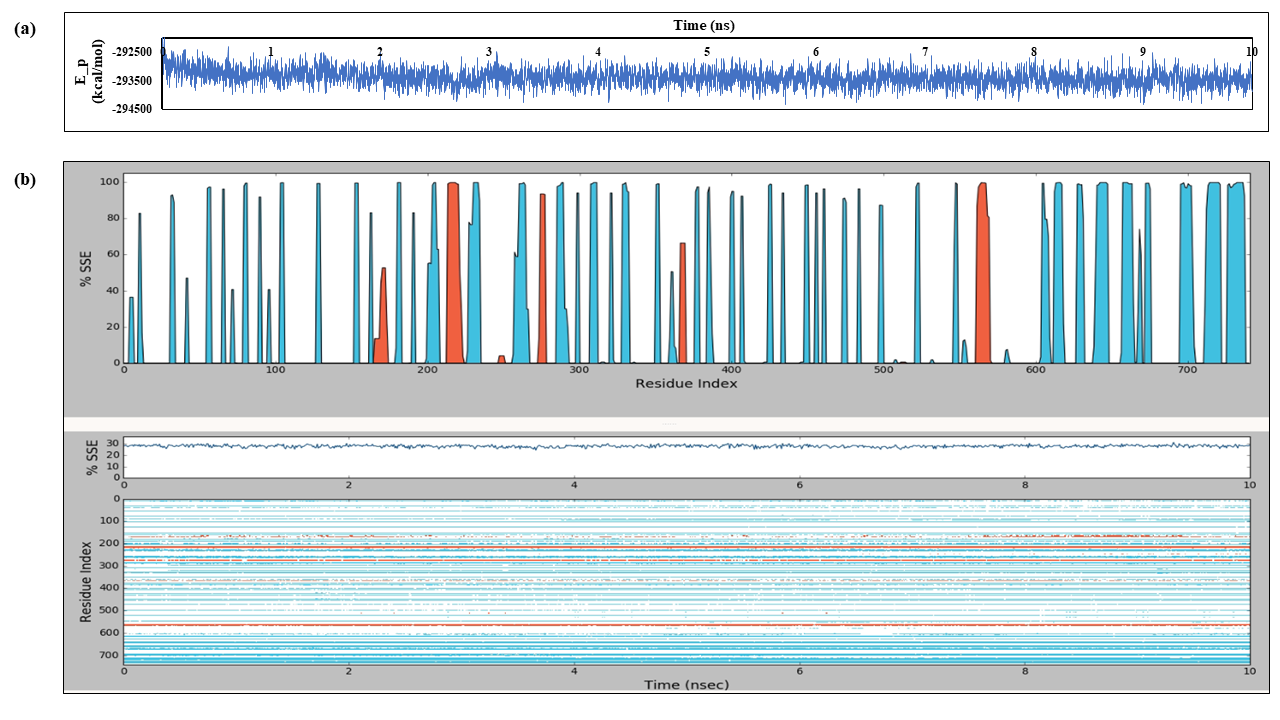


**
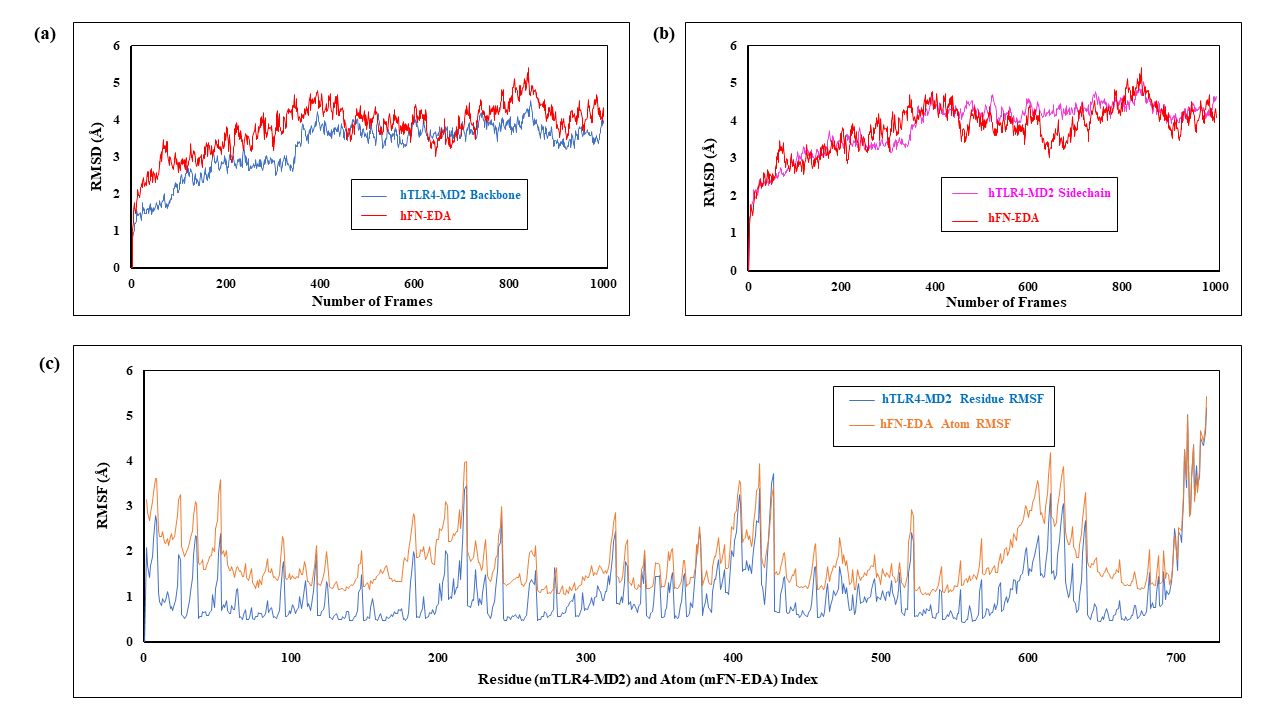
Figure S30: MD simulation outputs for human TLR4-MD2-FN-EDA. (a) E_p (potential energy) plot vs simulation time; (b) TLR4-MD2 secondary structure changes during simulation where light blue refers to β-strands and orange color refers to α-helices; (c) hTLR4-MD2 Backbone and hFN-EDA RMSD plots; (d) TLR4-MD2 Backbone RMSD plot with FN-EDA ; (e) Backbone human RMSF plot.**

- Outputs for mutated Arg44 in mouse FN-EDA in TLR4-MD2-FN-EDA complex


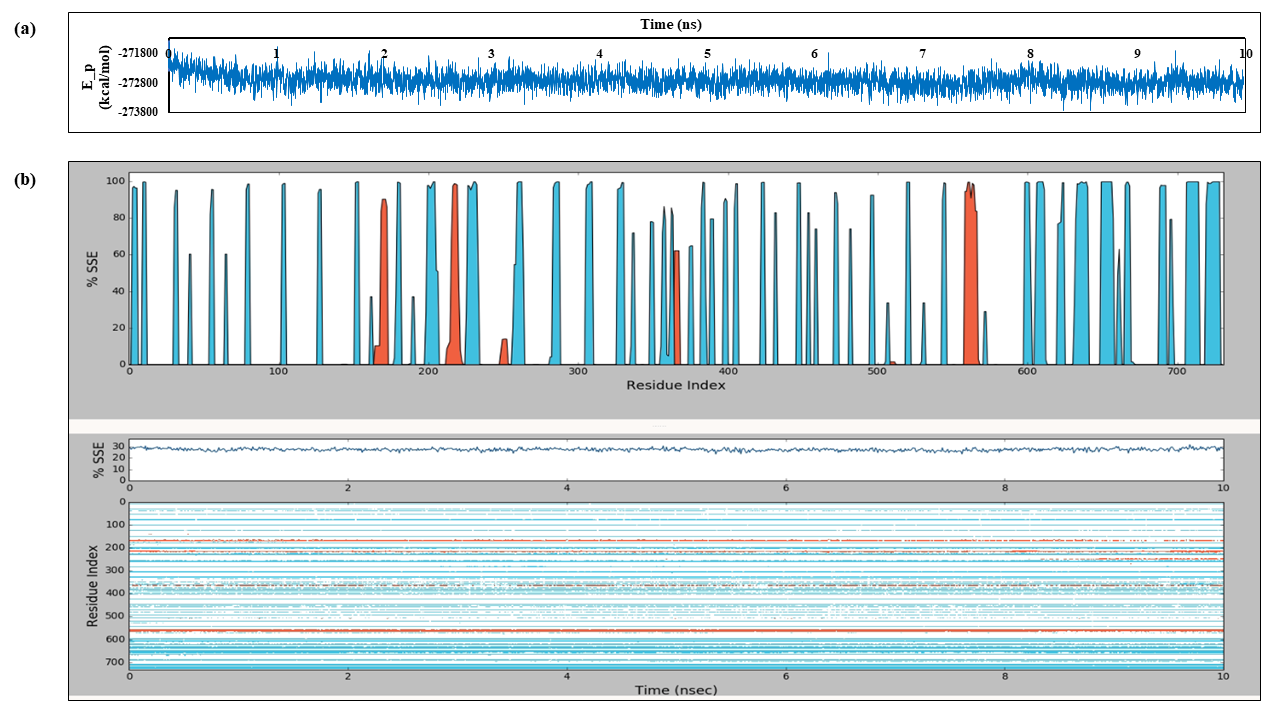


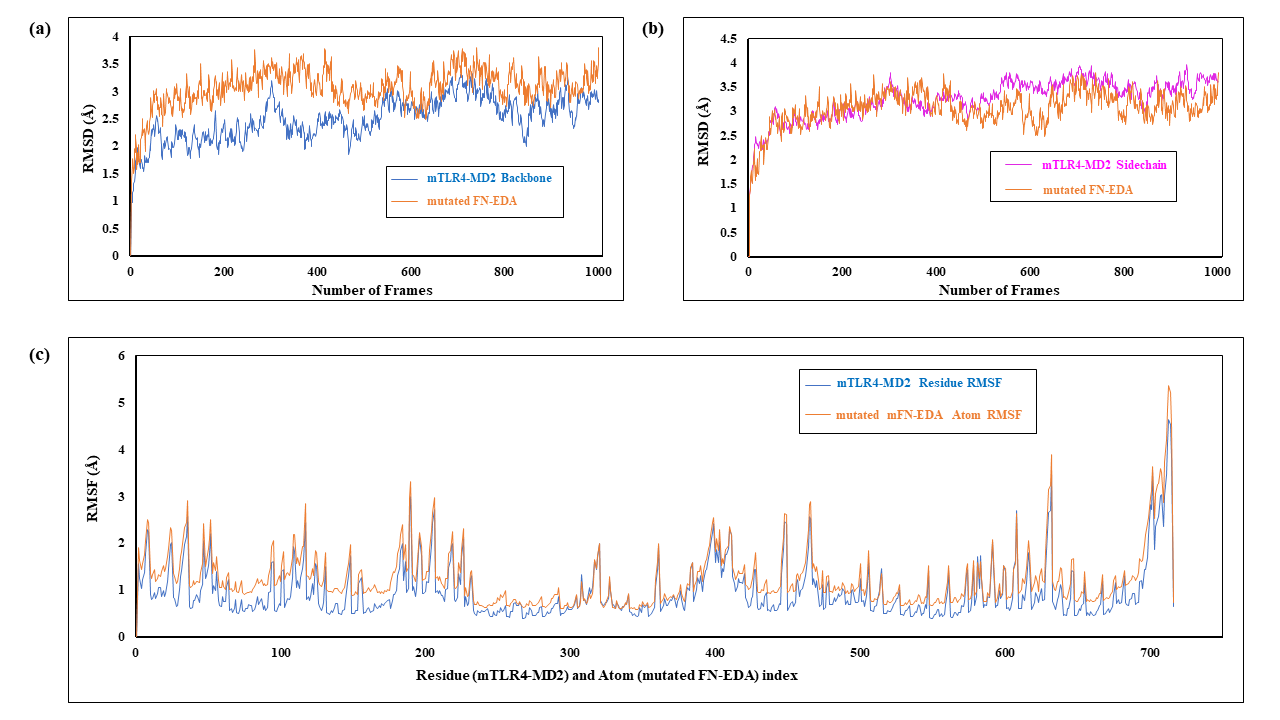


**Figure S31: MD simulation outputs for mutated Arg44 in mouse FN-EDA in TLR4-MD2-FN-EDA (a) E_p (potential energy) plot vs simulation time; (b) TLR4-MD2 secondary structure changes during simulation where light blue refers to β-strands and orange color refers to α-helices; (c) TLR4-MD2 Backbone RMSD plot with FN-EDA; (d) TLR4-MD2 Sidechain RMSD plot with FN-EDA; (e) Backbone RMSF plot**


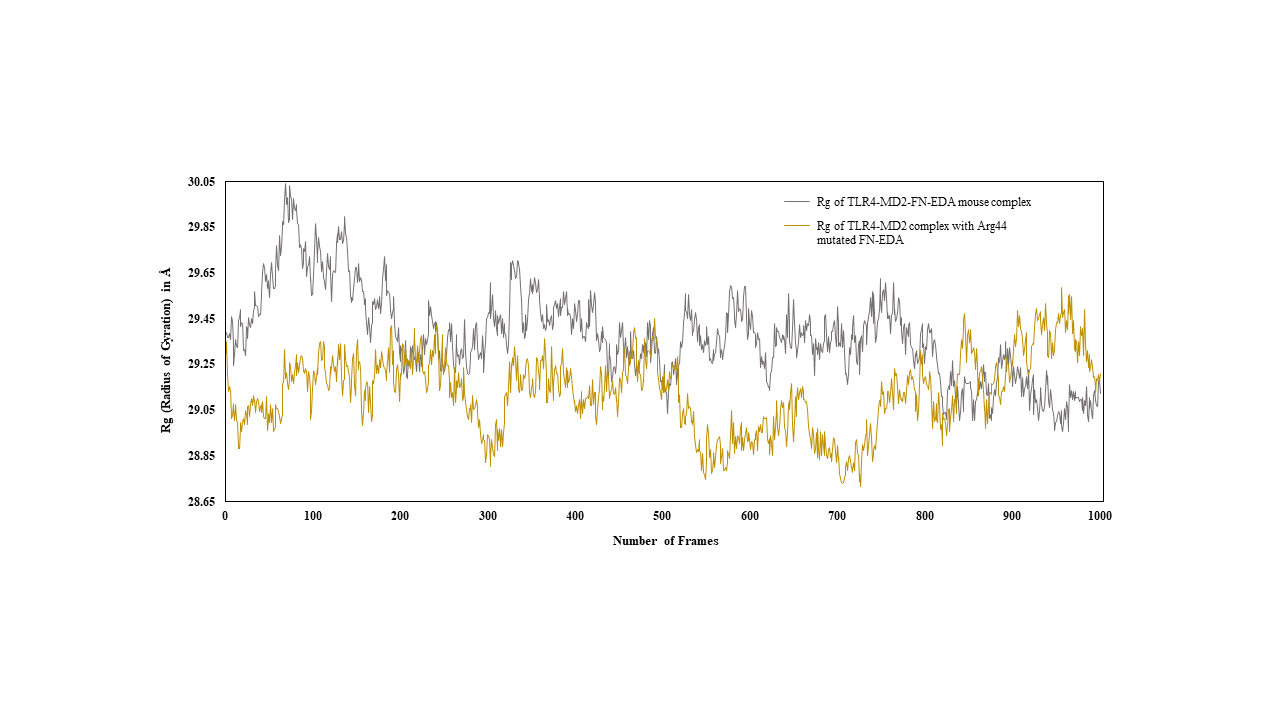


**Figure S32: Radius of gyration (Rg) plots of mutated (yellow) and non-mutated (grey) mouse TLR4-MD2-FN-EDA complex, where lower Rg values of mutated complex indicate compactness of the protein complex**
